# Identification of emerging viral genomes in transcriptomic datasets of alfalfa (*Medicago sativa* L.)

**DOI:** 10.1186/s12985-019-1257-y

**Published:** 2019-12-10

**Authors:** Peng Jiang, Jonathan Shao, Lev G. Nemchinov

**Affiliations:** USDA/ARS, Beltsville Agricultural Research Center, Molecular Plant Pathology Laboratory, Beltsville, MD 20705 USA

**Keywords:** Alfalfa (*Medicago sativa* L.), Transcriptomic discovery, Plant viruses

## Abstract

**Background:**

Publicly available transcriptomic datasets have become a valuable tool for the discovery of new pathogens, particularly viruses. In this study, several coding-complete viral genomes previously not found or experimentally confirmed in alfalfa were identified in the plant datasets retrieved from the NCBI Sequence Read Archive.

**Methods:**

Publicly available *Medicago* spp. transcriptomic datasets were retrieved from the NCBI SRA database. The raw reads were first mapped to the reference genomes of *Medicago sativa* and *Medigago truncatula* followed by the alignment of the unmapped reads to the NCBI viral genome database and de novo assembly using the SPAdes tool. When possible, assemblies were experimentally confirmed using 5′/3′ RACE and RT-PCRs.

**Results:**

Twenty three different viruses were identified in the analyzed datasets, of which several represented emerging viruses not reported in alfalfa prior to this study. Among them were two strains of cnidium vein yellowing virus, lychnis mottle virus and *Cactus virus X*, for which coding-complete genomic sequences were obtained by a de novo assembly.

**Conclusions:**

The results improve our knowledge of the diversity and host range of viruses infecting alfalfa, provide essential tools for their diagnostics and characterization and demonstrate the utility of transcriptomic datasets for the discovery of new pathogens.

## Background

Alfalfa (*Medicago sativa* L.) is one of the most extensively cultivated forage legumes in the world [1]. It has recently become the third most valuable field crop in the United States with an estimated worth of over $9.3 billion, which is $1.2 billion more than that of wheat, according to the National Alfalfa and Forage Alliance [2]. Alfalfa productivity is often limited by various biotic and abiotic components in the ecosystem [1, 3, 4]. With the increase in the production of monocropped alfalfa, infectious diseases, including viruses, have became more common. Traditionally, viral infections of alfalfa have been considered by producers, breeders, growers and research communities as diseases of limited importance. Nonetheless, they are widespread in major alfalfa cultivation areas and their contribution to the severity of complex infections involving multiple pathogens is poorly understood. Recently, many emerging viral diseases of alfalfa have been described that have the potential to cause serious yield losses. These include a rhabdovirus that was diagnosed in alfalfa plants displaying multiple abnormalities [5]; a new enamovirus from Argentina, *Alfalfa enamovirus-1* [6] (AEV-1) that was detected in alfalfa plants showing dwarfism symptoms; an AEV isolate from Sudan, designated AEV-2 [7]; *Alfalfa virus S*, a new species of the family *Alphaflexiviridae* discovered in alfalfa samples exhibiting chlorosis and stunting [8]; alfalfa virus F, a new member of the genus *Marafivirus* [9]; and *Alfalfa leaf curl virus* found in plants displaying leaf curling symptoms [10]. Alfalfa appears to be widely infected with seed-transmitted partitiviruses and the biological significance of these in alfalfa is currently unknown and requires further investigation [11, 12].

Publicly available transcriptomic datasets have became a valuable tool for the discovery of new pathogens, particularly viral sequences [11, 13–16]. The retrieval of complete or nearly complete viral genomes from transcriptomic data improves our knowledge of the diversity and host range of these pathogens and provides essential tools for their diagnostics and characterization. In this study, we performed a systematic survey of alfalfa transcriptomic datasets publicly available at NCBI. The survey indicated that approximately 90% of *Medicago sativa* samples employed in the generation of the deposited datasets contained viruses. Several emerging viruses were identified that had not been reported to infect alfalfa prior to this study or had not been experimentally confirmed in the plant.

## Methods

Viral sequences were identified in alfalfa datasets retrieved from the NCBI Sequence Read Archive (https://www.ncbi.nlm.nih.gov/sra). The raw sequencing reads were first mapped to the reference genomes of *Medicago sativa* (http://www.medicagohapmap.org/downloads/cadl) and *Medicago truncatula* (http://www.medicagogenome.org/) with the Bowtie2 software described below. Those reads that did not map to the reference genomes were assembled into contigs using SPAdes (v.3.12.0) and searched (BLASTn with default setting and the following parameters: 20 threads, two alignments and two descriptions) against other plant genomes (rice, apple, beet, arabidopsis and tomato) to check for possible cross-run contamination. Reads that did not map to any plant species were aligned to the NCBI viral genome database (https://www.ncbi.nlm.nih.gov/genome/viruses/). The alignments were performed using BBMap (v. 37.66), SeqMan Ngen (version 15.2.0, build number 130; mer size 17–21, minimum match percentage 85%) and Bowtie 2 (v.2.3.4) with the following very sensitive settings: -D 20 -R 3 -N 0 -L 20 -i S, 1, 0.50 and -p 20 (threads). In general, the very sensitive settings increase the search effort of Bowtie2 (D, R) by increasing the cutoffs for which Bowtie would stop searching. The length of the seed substring (L) was shortened to 20 from 22 and the interval (i) between substrings was decreased to 0.50. The reads that were mapped to the reference viral sequences related to the viruses of interest and to the assembled viral contigs from the datasets, were sequestered and assembled de novo using SPAdes (v.3.12.0) [17] with the following parameters: kmers 21,31,41,51,81,91,95, −m 800 -t 20. Wet lab experiments to confirm the identified viral sequences were performed only with *Medicago sativa* amalgavirus 1 (MsAV1). Plant materials infected with other viruses were not available for experimental confirmation of the transcriptomic data. For the MsAV1 experiments, the same RNA samples employed in Nemchinov et al. [18] were used. Total RNA was extracted as previously described [18]. The 5′ end and 3′ end of MsAV1 were amplified using the SMARTer RACE 5′/3′ Kit (Takara Bio USA, Inc., Madison. WI). The primers used for 5′/3′ RACE and the specific RT-PCR detection of the virus are listed in the Additional file 1. Phylogenetic trees were constructed using MEGA 7 software [19] by applying maximum likelihood method (with 1000 replicates) based on the JTT matrix-based model. The protein sequences were aligned by Cluster W incorporated into MEGA7. The sequence pairwise identity analysis of MsAV1 RdRp sequences was performed using the MAFFT program of the Sequence Demarcation Tool (http://web.cbio.uct.ac.za/~brejnev/). The SIAS tool that calculates pairwise sequence identity and similarity from multiple sequence alignments (http://imed.med.ucm.es/Tools/sias.html) was used to predict amino acid identities between the Pro-Pol regions of cnidium vein yellowing virus (CnVYV) and lychnis mottle virus (LycMoV).

## Results

**Fig. 1:**
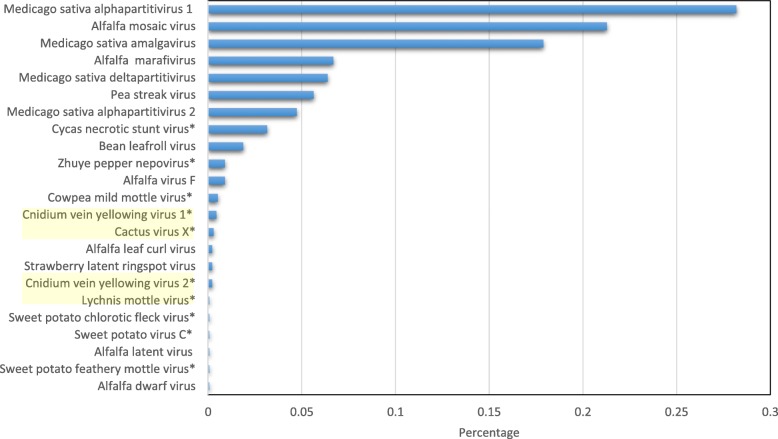
Virome heterogeneity found in 655 publicly available alfalfa transcriptomic datasets. * indicates viruses not previously reported in alfalfa. Highlighted are viruses that were characterized in this study

We performed a systematic survey of 655 alfalfa transcriptomic datasets publicly available from the NCBI. The survey indicated that approximately 90% of *Medicago sativa* samples employed in the generation of the deposited datasets contained viruses. Twenty three different viruses were found in these transcriptomes, including several viruses not previously reported in alfalfa. The heterogeneity of the viral contigs identified in this study is summarized in Fig. 1 (see Additional file 2 for details). Essentially complete viral genomes were assembled for three emerging alfalfa viruses that have not been identified in alfalfa prior to this study (CnVYV, LycMoV and *Cactus virus X*) and for one virus that had not been experimentally confirmed in the plant (MsAV1).

### *Medicago sativa* amalgavirus 1 (MsAV1)

The *Medicago sativa* amalgavirus 1 sequence was originally reported by Wang and Zhang in 2013 under the GenBank accession GAFF01077243.1 and later dubbed MsAV1 by Nibert et al. [20]. Prior to this study, the virus was not found in the US and its sequence had not been experimentally confirmed.

**Fig. 2:**
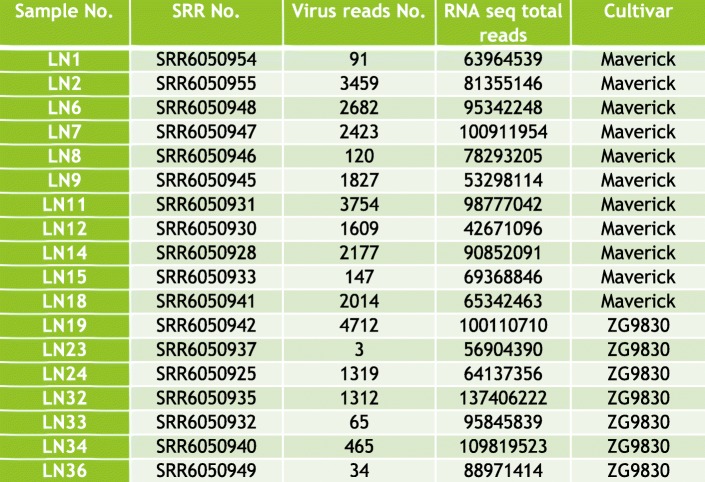
Medicago sativa amalgavirus 1 (MsAV1) reads found in alfalfa datasets

**Fig. 3:**
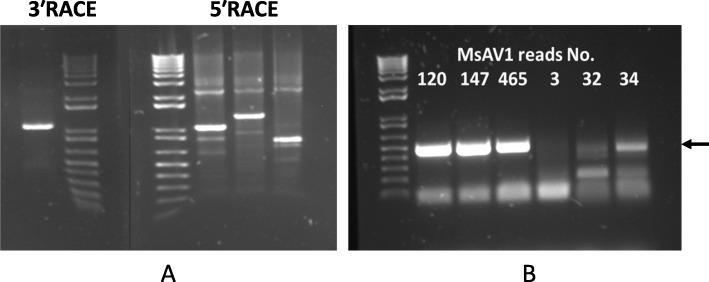
**a,** 3′ RACE and 5′ RACE PCR products generated from the MsAV1-infected tissues. Lane 1: 3′ RACE product amplified with a virus-specific primer GSP 2 and a 3′-CDS primer from the SMARTer RACE kit. Lanes 3,4, and 5: 5′RACE products amplified with different virus-specific primers (GSP1–1, GSP1–2 and GSP1–3, respectively) and a 5′-CDS primer from the SMARTer RACE kit; M: 1 kb Plus DNA Ladder (ThermoFisher Scientific, MA, USA). **b,** RT-PCR results obtained using primers MsAV1 F1 and MsAV1 R1 from selected alfalfa samples that had a low number of the MsAV1 reads. The arrow indicates RT-PCR amplicons of the expected size (496 bp)

**Fig. 4:**
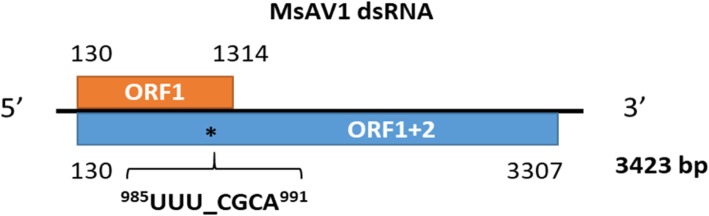
Schematic representation of the genome organization of Medicago sativa amalgavirus1 and the putative + 1 programmed ribosomal frameshifting motif in MsAV1

The RNA-seq data in which the virus reads were discovered, derived from the publicly available datasets SRR6050922 to SRR6050957 generated in our laboratory from the US alfalfa cultivars Maverick and ZG9830 [18]. The identified raw viral reads were mapped to the reference genome of MsAV1 (GAFF01077243.1; NC_040591.1) [20, 21] by Bowtie2 [22]. Each assembled transcriptome was individually searched for amalgavirus sequences using the BLAST tool [23]. The numbers of amalgavirus reads found in the datasets are listed in Fig. 2. Among the 36 screened alfalfa datasets described in details in [18], half included MsAV1 reads (Fig. 2). In addition, the assembled contigs were searched with BLASTX against a database of the RNA-dependent RNA polymerase (RdRp) motif sequences of known RNA viruses as described in Kim et al. [11] (2018). The resulting viral contigs were assembled into a complete viral genome**.** The 5′/3′-terminal sequences of the viral genome were determined experimentally by RACE (Fig. 3 a). The MsAV1 genome consisted of a single molecule of dsRNA that was 3423 nt in length and contained two putative overlapping open reading frames (ORF): ORF1, which encoded coat protein (CP) and ORF2, which encoded RNA-dependent RNA polymerase (RdRP) (Fig. 4). The 5′ UTR was 129 nt-long and a the 3′ UTR was 116 nt-long. The viral ORF1 (CP) was 1184 nt-long and a ORF2 (RdRP) was 3177 nt-long. The putative + 1 ribosomal frameshifting motif of MsAV1 was UUUCGCA and was found at the nucleotide positions 985–991 (Fig. 4) suggesting that the viral RdRp encoded by ORF2 is expressed as a fusion protein via a ribosomal frameshift mechanism [20]. RT-PCR with virus-specific primers designed based on the in silico-generated sequence confirmed the virus identity (Fig. 3 b).

**Fig. 5:**
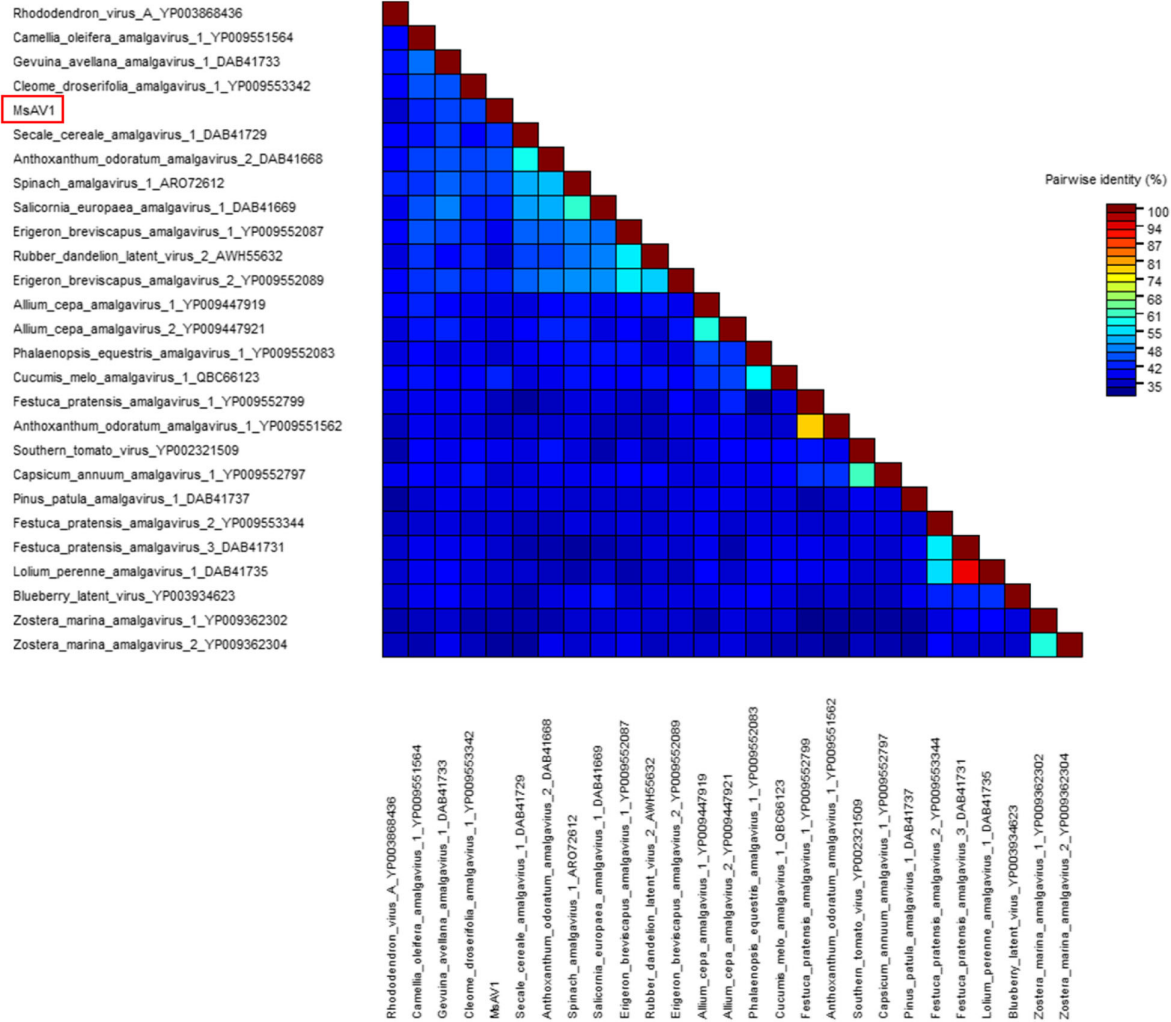
The pairwise identity plot of RdRps in the family *Amalgaviridae*. The alignment was performed using MAFFT software and visualized by the Sequence Demarcation Tool (http://web.cbio.uct.ac.za/~brejnev/)

**Fig. 6:**
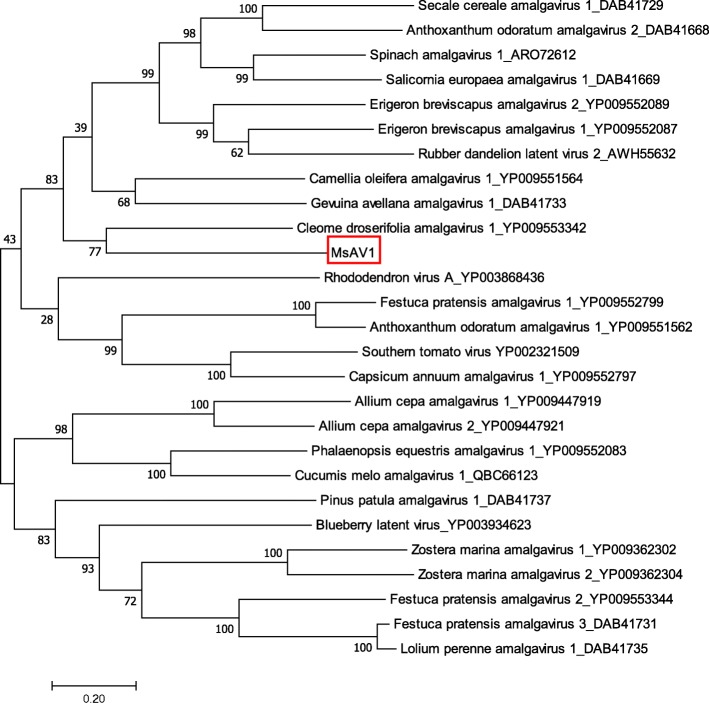
Phylogenetic relationships between MsAV1 and other members of the family *Amalgaviridae*, based on the alignment of the amino acid sequences of their RdRPs. The trees were constructed using the Maximum Likelihood method of MEGA 7 [19] with 1000 bootstrap replicates

Pairwise comparison of the MsAV1 RdRP sequences with other sequences in the family *Amalgaviridae* showed that RdRp is highly diverse (Fig. 5). Phylogenetic results based on the alignment of MsAV1 RdRP with that of other amalgaviruses suggested that MsAV is more related to the cleome droserifolia amalgavirus 1 (YP009553342), unclassified member of the family *Amalgaviridae* [20] (Fig. 6). The U.S. isolate of MsAV1 was 100% identical to the isolate GAFF01077243.1/NC040591 from China [20, 21] at both the nucleotide and amino acid levels, indicating the same origin of the virus. This is the first experimental confirmation of MsAV1 infection in alfalfa in the United States, which is significant because amalgaviruses are known to be vertically transmitted through seeds.

### Cnidium vein yellowing virus

Cnidium vein yellowing virus (CnVYV) is a bipartite, linear positive-sense ssRNA virus, a tentative member of the family *Secoviridae*, order *Picornavirales* [24]. Two isolates of the virus, CnVYV-1 and CnVYV-2, were found to infect cnidium plants (*Cnidium officinale*) in Korea [24]. To the best of our knowledge, no other hosts for CnVYV have been reported. Currently, the virus is not listed by ICTV as either an established or an unassigned species [25, 26].

For this study, the datasets were retrieved from NCBI Sequence Read Archive (SRA) and the contigs were individually assembled as described in the Materials and Methods. A total of 603,368 out of 108,082,074 (0.55%) raw Illumina pair-ended reads were found in accessions SRR2089795 and SRR2089796 of the BioProject PRJNA289195 [27] that mapped to the genome of CnVYV (GenBank accessions numbers: KR011028, KR011029, KR011030 and KR011031). When both datasets (SRR2089795 and SRR2089796) were checked for possible cross-run contamination, no plant species other than *Medicago* spp. were detected: 96% of the contigs had BLAST hits that corresponded to either *M. sativa* or *M. truncatula*.

The virus reads from both accessions SRR2089795 (284,134 reads out of 51,471,740) and SRR2089796 (342,594 reads out of a total of 56,610,334 reads) were assembled into coding-complete bipartite viral genomes, consisting of RNA1 and RNA 2 segments from two different strains of CnVYV-related virus. The virus strains were provisionally designated CnVYV-A1 and CnVYV-A2.

The genome of CnVYV-A1 assembled from the accession SRR2089795, had two RNA molecules of 6983 and 3588 nucleotides in length, respectively, excluding the poly (A) tails (see Additional file 3). At the nucleotide level, the RNA 1 segment of the CnVYV-A1 had ~ 75.6% identity (coverage 71%, E value = 0.0;) to RNA1 from the CnVYV-1 isolate (KR011028.1), which was the top BLAST hit for the CnVYV-A1.

The RNA1 segment of the CnVYV-A1 strain contained a single ORF encoding for a putative polyprotein of the 2201 amino acids long (P1). The P1 had 79.6% amino acid identity to the polyprotein 1 of CnVYV-1 (AKN59243.1). Other top BLAST hits included polyproteins from the CnVYV-2 isolate (AKN59245.1) (79.07% identity, coverage 100%, E value = 0.0), lychnis mottle virus (LycMoV), (coverage 100%; E-value = 0.0; identity 77.5%; accession AKN59247.1) and strawberry latent ringspot virus (SLRSV), (coverage 100%; E-value = 0.0; identity 77.6%; accession YP_227367.1). Thus, BLAST searches based on the amino acid alignments of P1 indicated the close relationship of the CnCVYV-A1 not only to the reference CnCVYV strains (1 and 2), but also to LycMoV and SLRSV. LycMoV is a tentative member of the family *Secoviridae* and SLRSV is an unassigned species in the same family [25].

The RNA 2 segment of the CnVYV-A1 sequence retrieved from the accession SRR2089795 was 77.03% identical to the RNA 2 from LycMoV isolate Andong [28] (coverage 89%, E-value = 0.0; KR011033.1) at the nucleotide level. CnVYV-2 was the second closest match with a percent identity of 77% (coverage 58%, E value = 0.0; KR011031.1). RNA2 encoded a polyprotein that was 994 aa-long (P2) which, based on the BLASTP search, was most closely related to LycMoV (coverage 100%; E-value = 0.0; identity 92.45%; accession AKN59248.1). Other top hits included SLRSV (coverage 100%; E-value = 0.0; identity 70.2%; YP_227368.1) and CnVYV-2 (coverage 80%; E-value = 0.0; identity 88.5%; AKN59246.1).

The numbers were slightly different for the CnVYV-A2 strain, which was retrieved from the accession SRR2089796 (see Additional file 3). The RNA1 segment of the CnVYV-A2 strain was 6892 nt long, excluding the poly(A) tail. At the nucleotide level, it was 71% identical to the complete RNA 1 of the CnVYV-1 Yeongyang isolate (coverage 71%; E-value = 0.0; KR011028.1). RNA1 of the CnVYV-A2 strain encoded a polyprotein of 2201 aa-long that was 78.9% identical to that of the CnVYV-1 Yeongyang isolate (coverage 100%; E-value = 0.0; AKN59243.1). Other top BLAST hits included LycMoV (coverage 100%;E-value = 0.0; identity 77.53%; accession AKN59247.1) and CnVYV-2 (coverage 100%; E-value = 0.0; identity 78.89%; AKN59245.1). The nucleotide and polyprotein sequences of the RNA 1 segments of the CnVYV-A1 and CnVYV-A2 strains were 90.6 and 96.6% identical, respectively.

The RNA 2 of the CnVYV-A2 strain, which was retrieved from the accession SRR2089796 was 3611 nucleotides long, excluding the poly(A) tail. It was 77.29% identical to the RNA 2 of the CnVYV-2 Yeongyang isolate (coverage 57%, E-value = 0.0; accession KR011031.1). Other BLAST hits (LycMoV and CnVYV 1) had a low coverage (1%). The RNA 2 translated into a polyprotein of 994 aa-long, which, similar to the P2 in CnVYV-A1, showed a top BLAST hit for LycMoV (coverage 100%; E-value = 0.0; identity 90.54%; accession BBE07891.1). Other closely related species were SLRSV (coverage 100%; E-value = 0.0; identity 70.01%; YP_227368.1) and CnVYV 2 (coverage 80%; E-value = 0.0; identity 87.73%; AKN59246.1). The RNA 2 segments of the CnVYV-A1 and A2 strains were 90.7 and 97.9% identical at the nucleotide and amino acid levels, respectively.

**Fig. 7:**
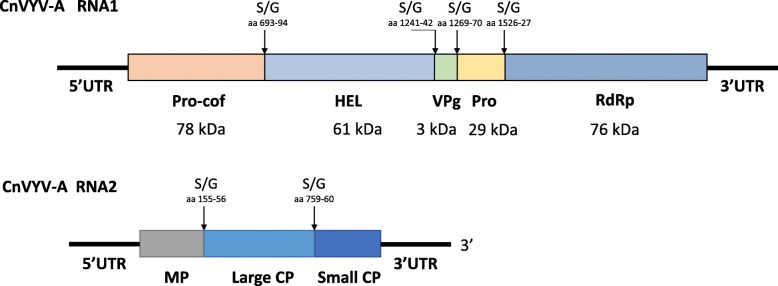
Putative genomic organization of the alfalfa strains of cnidium vein yellowing virus (CnVYV-A). The open reading frames are indicated by boxes and the putative serine/glycine (S/G) cleavage sites and their amino acid positions are indicated by arrows

A Pfam database search (version 32.0) [29] of the CnVYV-A strains identified two conserved domains in their P1: RNA helicase (PF00910; E-value = 2.1e-28) and RdRp (PF00680; E-value = 1.5e-60). The putative cleavage sites between these proteins were all of the serine/glycine type (S/G), as was observed in the CVYV-1 and CVYV-2 isolates. InterPro scan (version 5.36–75.0) [30] predicted two CP domains typical for RNA 2 polyproteins (IPR029053) in P2 of the CnVYV-A. The SIAS tool [31] detected several conserved domains in P2 of the CnVYV-A strains that were characteristic of CP(s) signatures found in secoviruses. Based on these predictions, we anticipated that CnVYV-A strains share a similar genome organization with other closely related viruses of the family *Secoviridae* **(**Fig. 7).

We thus concluded that CnVYV-A1 and CnVYV-A2 represent isolates of the same virus strain adapted to alfalfa, for which we propose the name CnVYV-A (alfalfa).

### Lychnis mottle virus

Lychnis mottle virus (LycMoV) is a tentative member of the family *Secoviridae* that was first reported in *Lychnis cognata*, a flowering plant in the family *Caryophyllaceae* [28]. In 2017, the virus was also isolated from the leaves of *Vincetoxicum acuminatum* in Japan and the complete nucleotide sequence of LycMoV-J was obtained [32].

A total of 400,676 out of 56,610,334 (0.7%) raw Illumina paired-end reads were found in the accession SRR2089796 [27] that mapped to the genome of the LycMoV isolate Andong (GenBank accessions numbers KR011032 and KR011033). The genome of the alfalfa isolate of LycMoV (LycMoV-A) that was assembled from the accession SRR2089796, consisted of two segments of 6972 and 3584 nucleotides in length (corresponding to RNA1 and RNA 2, respectively), excluding the poly (A) tails and appeared to be coding-complete (see Additional file 3).

BLAST searches showed that the RNA1 segment of LycMoV-A had 74% identity to that of the LycMoV Andong isolate (coverage 79%; E-value = 0.0; KR011032.1). SLRSV was the second hit with a low query coverage (22%). However, when the RNA 1 was translated into a putative polyprotein 2201 aa in length and analyzed by BLAST, the top hit was CnVYV-1 (score 3631; coverage 100%; E-value = 0.0; identity78.70%; accession AKN59243.1). LycMoV was the second highest hit (score 3615; coverage 100%; E-value = 0.0; identity 79.07%; BBE07890.1). A translation of the nucleotide sequence presumably corresponding to LycMoV, into an amino acid sequence resembling that of the CnVYV suggested that CnVYV and LycMoV represent the same viral species.

The RNA 2 segment of LycMoV-A was 77.3% identical to that of the LycMoV Andong isolate at the nucleotide level (coverage 89%; E-value = 0.0; KR011033.1). At the amino acid level, P2 had 90.9% percent identity with that of the LycMoV-J isolate (coverage 100%; E-value = 0.0; accession BBE07891.1). The Andong isolate of LycMoV (92.3% identity; AKN59248.1) was the second highest hit and SLRSV (70.1% identity; YP_227368.1) was the third highest.

### Relationship between the CnVYV strains and LycMoV

**Fig. 8:**
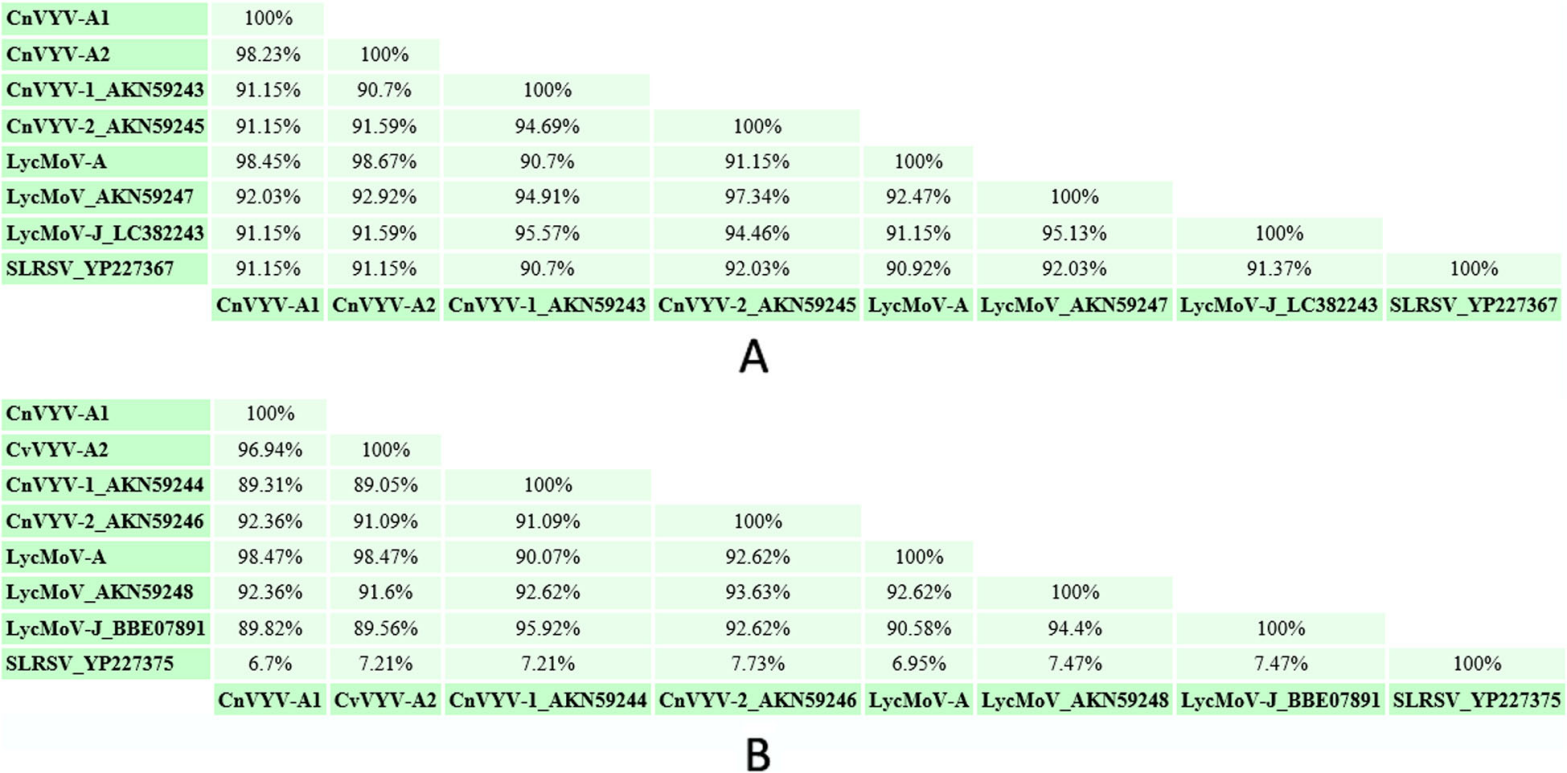
Amino acid identities between the Pro-Pol **a** and CP **b** regions of the CnVYV-A strains, the CnVYV1 and CnVYV2 strains, LycMoV-A, LycMoV, LycMoV-J and SLRSV, as predicted by the SIAS tool [31]

To determine whether LycMoV and CnVYV are strains of the same virus species or correspond to the distinct species as was suggested by Yoo et al. [24, 28] for the Yeongyang isolates of CnVYV from *Cnidium officinale* and the Andong isolate of LycMoV from *Lychnis cognata*, we compared the amino acid identities between their Pro-Pol and CP regions, which are currently used as species demarcation criteria by the ICTV [25]. The SIAS tool [31] predicted the following amino acid identities between the Pro-Pol regions of these viruses (Fig. 8**).** Although the identities were slightly different from the ones generated by BLASTP due to the different formula used for the calculations, all the conserved Pro-Pol values were higher than 80% (the ICTV criteria for species demarcation are less than 80% identity) and all the CP values, except that for SLRSV, were higher than 75% (ICTV criteria for species demarcation are below 75% identity). We speculate that CnVYV-A, together with all the other viruses in this study group (CnVYV-1, CnVYV-2, LycMoV-A, LycMoV and LycMoV-J) and with the exception of SLRSV due to the low identity of its CP region, constitute individual strains of the same viral species isolated from different hosts, for which a collective name reflecting its biological characteristics and taxonomic position is required. We suggest the following temporary name for the species: cnidium vein yellowing-like virus (CnVYLV).

**Fig. 9:**
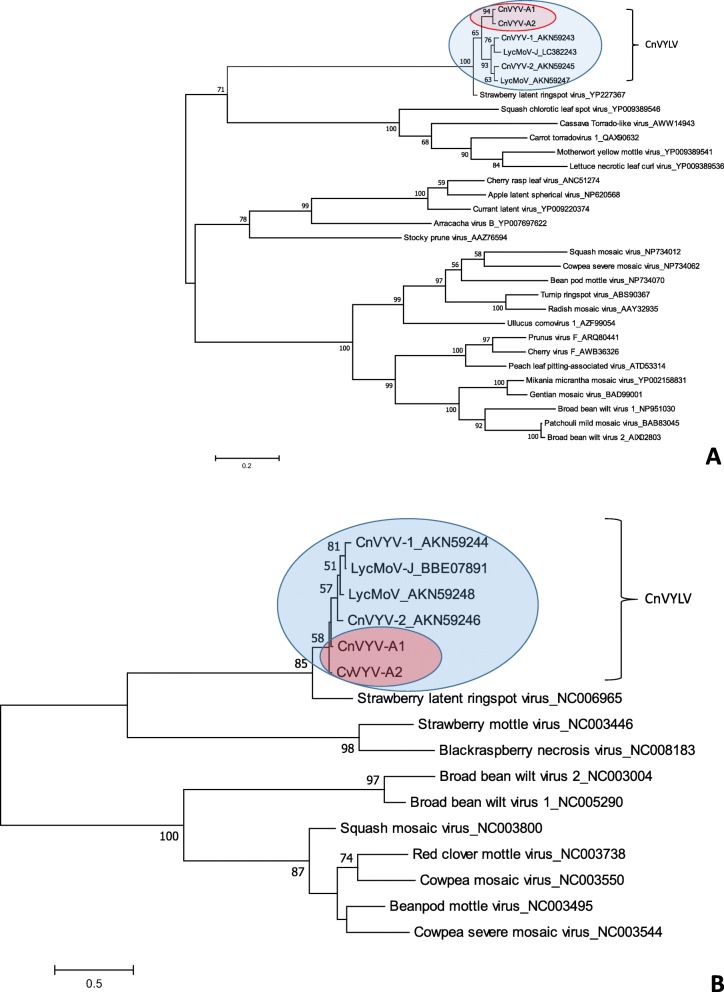
Phylogenetic analyses based on the amino acid alignments of the predicted Pro-Pol region of RNA1 **a** and the CP region of RNA 2 **b** of the CnVYV-A strains and other members of the family *Secoviridae*. The trees were generated using the Maximum likelihood algorithm of MEGA7 [19] with 1000 bootstrap replicates. Blue oval indicates different viral strains that belong to the proposed species tentatively named cnidium vein yellowing-like virus (CnVYLV). Red oval indicates viral strains found in alfalfa

Phylogenetic analyses based on the alignments of the Pro-Pol region between the protease CG motif and the RdRp GDD motif (CG/GDD) in RNA1 and the predicted CP region in the RNA 2 segment supported this conclusion: CnVYV-A1 and CnVYV-A2 were grouped together with the reported strains of the CnVYV and LycMoV (Fig. 9) and the cluster branched out toward a more diverse strawberry latent ringspot virus.

### Cactus virus X

*Cactus virus X* (CVX) is a member of the family *Alphaflexiviridae*, genus *Potexvirus*. It infects many species in the plant family *Cactaceae* worldwide [33]. Prior to this report, it had not been identified in alfalfa. The NCBI reference sequence for CVX was reported under two identical accessions NC_002815.2 and AF308158 [34].

In this work, we have detected 2028 paired-end CVX-related reads in the publicly available alfalfa transcriptomic dataset SRR7751381 from the NCBI BioProject PRJNA487676 [35] . The raw reads were assembled de novo into a coding-complete virus genome consisting of a single molecule of linear ssRNA 6603 nucleotides in length, excluding the poly(A) tail (see Additional file 3). At the nucleotide level, the alfalfa isolate of CVX (CVX-A) was 97% identical to the CVX reference sequence (coverage 100%; E-value = 0.0; AF308158.2). Other BLAST hits included different CVX isolates with lower alignment scores.

The 5’UTR of the CVX-A consisted of 74 nucleotides preceding the initiation codon of ORF1 and began with the sequence CCAACACCAA, which is most likely missing a few nucleotides (~ 5 nt) at the very 5′ terminus. The 3’UTR consisted of 99 nucleotides downstream of the termination codon of the ORF5 (pos. 6502–6504) and appeared nearly complete, missing at most one or two nucleotides. Viral RNA translated into five putative ORFs encoding for RdRp (ORF1, 75–4703 nt); triple gene block protein 1 (TGBp1; ORF2, 25 kDa; 4703–5392 nt); partially overlapping with the ORF2 triple gene block protein 2 (TGBp2; ORF3; 5355–5687 nt; 11.9 kDa); triple gene block protein 3 overlapping with the ORF 3 (TGBp3; ORF4; 5617–5811 nt; 6.7 kDa); and a putative coat protein separated from the ORF 4 by a 15 nt-long intergenic region (ORF5; 5827–6504; 24.3 kDa).

**Fig. 10:**
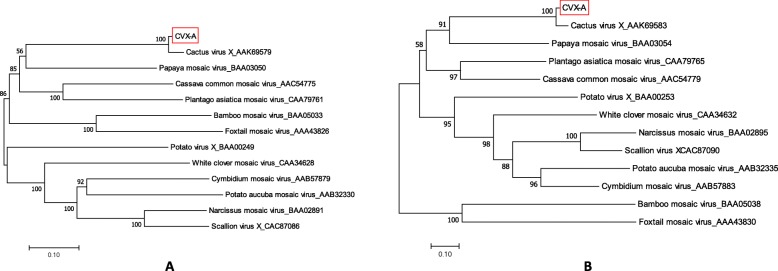
Phylogenetic analyses based on the amino acid alignments of the predicted RdRP **a** and the CP sequences **b** of CVX-A and other members of the family *Potexviridae*. The trees were generated using the Maximum likelihood algorithm of MEGA7 [19] with 1000 bootstrap replicates

We predicted that the genome organization of the CVX-A largely resembles that of the CVX [33]. BLASTP analysis of the aa identities of the putative CVX-A proteins with the corresponding proteins of the reference genome suggested their close relationship (Table 1). Phylogenetic analysis performed with the aa sequences of CVX-A RdRp and CP placed CVX-A in the same subcluster as CVX. We concluded that CVX-A represents a strain of CVX, adapted to alfalfa (Fig. 10).

**Table 1:** BLASTP alignment scores of the CVX-A and CVX

Alignment scores	RdRp	TGBp1	TGBp2	TGBp3	CP
Percent identity	98.2%	100%	98.18%	98.44%	100
E-value	0.0	0.0	1e-71	4e-36	7e-163
Query cover	100%	100%	100%	100%	100
Accession #	BAU68240.1	NP_148781.1	AIW81551.1	AMX81286.1	BAO73885.1

## Discussion

Publicly available transcriptomic datasets are becoming an increasingly valuable tool for the discovery of new pathogens, particularly viral sequences. In this study, we performed a systematic survey of 655 alfalfa transcriptomic datasets publicly available at the NCBI. Twenty three different viruses were found in the analyzed transcriptomes, including several emerging viral pathogens not previously reported in *M. sativa* prior to this study. Among them were two strains of cnidium vein yellowing virus, lychnis mottle virus and *Cactus virus X*, for which coding-complete genomic sequences were obtained by a de novo assembly. Based on the amino acid identities of the conserved Pro-Pol region, the CP sequences and the phylogenetic analyses, we suggested that CnVYV-A1, CnVYV-A2, CnVYV-1, CnVYV-2, LycMoV-A, LycMoV, and LycMoV-J represent different strains of the same viral species, tentatively called cnidium vein yellowing-like virus (CnVYLV). We have also presented the first in silico and experimental identification of *Medicago sativa* amalgavirus 1 in US alfalfa germplasm, which is significant because amalgaviruses are known to be vertically transmitted through seeds. Although amalgaviruses are not considered pathogenic, they could be of economic importance due to their potential role in mixed infections [36] and ubiquitous presence in some cultivars, species or genera of plants [37].

The reported results improve our knowledge of the diversity and host range of viruses infecting alfalfa and provide essential tools for their diagnostics and characterization.

## Conclusions

The systematic survey of alfalfa transcriptomic datasets publicly available at the NCBI indicated that approximately 90% of *Medicago sativa* samples employed in the generation of the deposited datasets contained viruses. Several emerging viruses were identified that had not been reported to infect alfalfa prior to this study or had not been experimentally confirmed in the plant. Coding-complete genomic sequences were obtained for cnidium vein yellowing virus (CnVYV), lychnis mottle virus (LycMoV) and *Cactus virus X* (CVX), all of which have not been diagnosed in alfalfa until now. Further research is required to confirm the in silico identification of these viruses and to determine their symptomatology, geographic distribution and economic importance to the alfalfa industry.

## Supplementary information

**Additional file 1:** Primers used in the 5′/3′ RACE and RT-PCR reactions for amplification of the MsAV1-derived products.

**Additional file 2:** Overview of viral contigs identified in 655 alfalfa transcriptomic datasets.

**Additional file 3: **Nucleotide sequences of the alfalfa strains of cnidium vein yellowing virus (CnVYV-A), lychnis mottle virus (LycMoV-A) and *Cactus virus X* (CVX-A).

## Data Availability

All nucleotide sequences described in the manuscript are available in the Additional file 3. The sequences have also been deposited to the Third Party Annotation Section of the DDBJ/ENA/GenBank databases under the accession numbers TPA: BK011041-BK011047.

## Abbreviations

CnVYV: Cnidium vein yellowing virus
CVX: *Cactus virus X*
LycMoV: Lychnis mottle virus
MsAV1: *Medicago sativa* amalgavirus 1
RACE: Rapid amplification of cDNA ends
RT-PCR: Reverse transcription polymerase chain reaction
