# Supplementary material for: Identification of emerging viral genomes in transcriptomic datasets of alfalfa (Medicago sativa L.)
Source: Virol J. 2019 Dec 10;16:153. doi: 10.1186/s12985-019-1257-y (PMC6902351; doi:10.1186/s12985-019-1257-y)
Supplement: Supplementary file 1 — Additional file 1: Primers used in the 5′/3′ RACE and RT-PCR reactions for amplification of the MsAV1-derived products. [file 12985_2019_1257_MOESM1_ESM.docx]

| Sequence Name | Amplicon type/size/position | Sequence |
| --- | --- | --- |
| MsAV1 F1 | Internal  496 bp  (1199-1218 nt) | CACGAACTTCAAGGGTGCAT |
| MsAV1 R1 | 496 bp  (1676-1695 nt) | GGTAGACCCTCCACACCAAA |
| MsAV1 GSP1-1 | 5ʹ RACE  990bp  (963-990 nt) | GCGAAAGGAACCAGCTTGCTTCTTCTCC |
| MsAV1 GSP1-2 | 5ʹ RACE  1238 bp  (1211-1238 nt) | CCGGCATTCCTTGACTTGTTATGCACCC |
| MsAV1 GSP1-3 | 5ʹ RACE  741bp  (714-741 nt) | GTTGTCAGGGCCTTCATAAAGCGCAACA |
| MsAV1 GSP2 | 3ʹ RACE  1019 bp  (2406-2432nt) | CGACCCCCTTACCATGTGACTAAAGTTC |

**Additional file 1**

**Primers used in the 5ʹ/3ʹ RACE and RT-PCR reactions for amplification of MsAV1-derived products**
