# Supplementary material for: Identification of emerging viral genomes in transcriptomic datasets of alfalfa (Medicago sativa L.)
Source: Virol J. 2019 Dec 10;16:153. doi: 10.1186/s12985-019-1257-y (PMC6902351; doi:10.1186/s12985-019-1257-y)
Supplement: Supplementary file 3 — Additional file 3: Nucleotide sequences of the alfalfa strains of cnidium vein yellowing virus (CnVYV-A), lychnis mottle virus (LycMoV-A) and Cactus virus X (CVX-A). [file 12985_2019_1257_MOESM3_ESM.pdf]

Additional file 3 (Jiang, Shao and Nemchinov, Virology Journal)

CnVYV-A1_RNA1

CTCCCAAGAAAAGCAATCTGCGAACTTTGTTACTAATTTGTTTTCTCAACTAGTTATTGGACTTATCCAATTTCAACAAT
CTTTTGCTCTTCTGTTTCAAGTGCTTTCTGTTGTGCTGCGGTAATACAGCTTTCCTTTTGGAATTCAAGTCTTAACTTGA
ACAACACCTTCTTTTCTTTATTTCTTCTTTCTTATCTTTTAGTAACCGCTTTAATTTTCCATGGGTTATTCAAGAGGTGC
GAGGGGGGGTTCTGATAAGGCAAAGCTTTGTGGAGTGTGCTTTGCTATCTTTCCTGATGTTCAGGAGAGAAAAGCACACT
ACAGGAAGTATAACTGCCGTGATGCATCTGGCATCCAGGGTGGTTTAGTGGTCACTGGGAAATGTGCTGCCCAGAAAGAG
AGGAATTTGAGCTCCTATTATGAAACTCAGGAGCTTCTTGCTGATGAAGAGGGTTCAATTGAGCAGATTGAGACCCAATT
CATGTCTGAGTTGCTGGAGTTTACCTCTGGCTACCCGGACGCAGGTTTTAGCTTTGGCACTATCAATGAGAAAGTGCCTA
AAATAGGTGCTTCTTACCCGGAGGCAATTTATCGTTCCTCGCTCTTTCATGCTGAGGAATATGCCAAGTGGCAATCACAT
AAACCTGGAGCAGCTGTAAATTTTCTTGCTCCTGATTATGTTCCACTTGCTGATGCTTGCATGAAAGGCGCTCGTTTCGC
CAAACCCCATGCTGGTCTTTGGGAAAGTAAGACGGATCGTACAGGTCTTGGCGGGGATGATATTAAATACCGTTTTGATA
ATGACGATCTTTTTGCCAAGACTGATCAGGGCCTCAGCAGATTTGTCTTTTCAGGCACTGTTAGGTCTGAGCTCCCGGCT
TTGCGGACGGTTCCAGATTTAACGCAAGCTGATATAAAAACCATGGCATCTGCTCTTTCTGATGAGTGGGTTGAATGGAT
TTATTTTGAGGAGGATTACGAGGAGTTTCTCTCGGATTCTTTTGAAGAATTCTATCCAGACGTCCCAGAGTCTCCTCTGG
AAGAAATTTTTAGCCCAGTAAGTGTCTTGGACTCAGCCAGCTCATACCTTCCTGGTACTAAGTGGTGTTATGATTCGGAT
ACAGAAGATTACCACCCTGTTGCGCCTATAATGAAGTATAGCAACAAAAAGTTTGGTTTGGACTTTGTTGATTATACTGA
TACAGAGTATTTGGAAGTGCCTGCTGGCAAGGTTTACACTGAGACCTGGTTTATAGATGGTCTGCTACCAGGTTTCTGGT
TTCTGCGTGGAACTCCCATAGATGAGGCAGTGGTGTGTGAAAAGCTCCCACTGATAGGGAAAACTCCCCTAGAGCTCATG
ATAGAATACCAGGTGCAACCTACTGGTGATCTTCTATTAGATATTCAAAGGGCTCTCTATGCTTATTATGAGCGTGTGGT
GAAACCCAATAAAAAATATTTTGACTTGAATGGGGTTCCCACATATCTTATTTTTGAGGACTGGAGAGCGGATTTGCGCT
TCCATTTGTTAGCACCATTTGTCCAAGGCAACCAACTGAAACATATGATGGTGATTATGAACAGTCCGCTTCCGGGCTGG
TTACTTGACTTCCCGAAAAGGCGTAAGGGAGGTGAAGTGCAAGCCAGTGGGTGGAATCCTTTCCGCTATTTTTCAAAACA
GGTGACGTTGGGCTTTCTGGATGGGATTGTGGCGAAACTCCAAGAATCACTTGGCTCGGTTATCTCTGCGCTTGGTTTTG
TATGGGATTTGATTATTAAGGCCAAGGATTATGTTTTCTCTTTGTTGGAGGATATTATTGCAAGAAAAGCGGATATCTTG
AAGGCATTGCTCCAACCTGTTCTGTATGTCTGTGGCTTTCTAACCTTTTTAGGGGCTATAAAGAGTCTCAAGTCTATTTT
GGAACACCACAAAATAGCACTTGACATTCTCACGACAGCGGCTGTGGGGTTGGGTGTTTATGTCATGGTAACATTCCTGA
AACAAGCTCACCTAGGGGCTATGAAAAGAGCTACTAGGGTCTGGGAGTTAGTTTGTGGCTGGGACAAGCCAACTGAGATG
AAGCAGGCTGATGAAAAGATCCTACAGACATTAGTGGAAGAGAACCCAGACCAAGAAGAGTGGCTGAAGGGCTTACTGGC
CTCTCCACACAGTGTGCCAGAGTGTGTTATAGATATAACGCACATGAAGGATCTTTTAAATCCCAAGTGTGCCACTTTTC
AAGAGGCCTTTGCTGTGGGTTCTACGGAAGCCTCCTCTGCTGGTATGCTATTTGGTTCAGGATTTATATGGAAGATACTT
CTCCTTCTTTGTCCCCTCTCCATGTTTGGAATCACTAAGACATTGTCCTGTGCTAAGGACTTGATTACAATCCAGGGTGG
ACAAGATGCTGCTGGTAGGTTCTTCCAGGAAGTTATTGGTGGTACCCAGGAGGTTTTCTACACCTTAACGGGTAGCAAAA
ATGAGTTTCTTGATTATATATATGCTACTGTAGGTGTGGATTTCCAGGCCTGGAGAGGAGAGGTTTTGGAGTTAACAACA
GCAACCCCAACCTCAATCTTTTTGGGACCACAAGAACGTTTGAAGCGTTTGAGAGCCTGTAAGGATAAAGCAGATCGGCT
GATACTCCAGATGGACTCTCGGAAAGTCCCAGGGGCTTATATCACACACTTTAACAATCTGCTTCAAAGCCTCGATCGTG
CTTTGGTGGAATGCCAGCAAGCTCTTTCTGTGGGTAAATGGCGGAAAACGCCTGCATGCATCTGGCTTTATGGAGATTCT
CATGTTGGGAAATCAGTTTGTACCCAATACCTGATTGATGATGTTCTAGACTCCCTTGATTATGCTCAAACTGGGAGAGT
CTTTTCCAGGAATGGTTCAGATTCTTTCTGGTCTTGCTATAAAAATCAAAGTGCAGTCTTGTATGATGATTTTGGAGCTG
TTTCTGAGGGTGGACACTTTGATGAGGCAGAGATAATACGTCTGATAGCTCCAGCTCCTCTCCCTTTAAATATGCCAAAT
TTGGAAGCAAAAGGGAATACTTGCTGCACCTCTGATTTCGTTTTTATAACGGCTAATCAGGCTGGCCTAACACCATCTGC
TGTTGTTCACTGTAAGAAAGCCTTTGAAAATCGAAGGCTCATTTTAGCTGAGGTTACTGCTGTTGAGGGTGGTCGTTACC
GTGACAGGTATCGTTTTACTATCCACCAGAGGAATGAACCATATGGACGGGATGAGCGCTTCCAAGCTATGAATTATGAA
CAGTTTCTCCAGTTCACAGTTAACCAATGCAAGCACCATTTTGAAGAACAAGTGGACTTGAGAGATGCCCCGCACACCCA
GATCTTTGATGCAGCTGATCAAATTGCGGCAGAAGCTGAGGAGGGGTTTCAGACAGCTGGAATAGATATTCCTTTTTGTA
TGCTCAAAAGTTTAGATGGGGATAAGATCACCTACTACTCACAGGAGTTGTTCAAGGAGATTACTGAAGACCTTTCTCCT
GAGGATGCTAGTATCATCCGTGCTCGTTTGGACCCGGGTGTTATAACACAGAGGATGGAGGCGTTATTGCCGAAGGCTGC
TTTTACTAATCTGAGAACACAGGTTGGTTTAGGTGTTGATCCTTTTGAGACACCCAACCCCTATTTTAATTGCACCCAAA

GGGCTAAAGTTGTGGGTCGTTTCATGAAGCCAAAAGTGTCGGAAGCACAAGTGCAGGAAAGTAAGAGAAGTTTTGGAGTG
ATAGTCAAAGAGTTGGTCAAGGGTGCATGTAAATCGCTAGCAGAAGCACCTTTTTTGGTAAAACTCATTCTTGGTTTTGG
GGCACTTTATTATGTAGGCTTGCCTATCCTTAGTTGGCTTAAGGAATTATATTCAGCCCCTTCACTCCTTACTTTATCTT
CTCTTGGTGTTGTCAGAGCTTCGGGTTCCCTCTCAAGTTCCCAGGATCAGGAGACAAGACGTACTGCCTCTGGTAGAGAG
CGGAGGCGCTATCTTTTGGAAGCATCAGGTCCTGGAGCACTTACAGCAAAGCCACAGGATGTTGAGACGGACCTCCGTTC
CCTCTCTAAGCACTTGGTTGGTTTTACTAGTATAGACTATCCGGATCACCACTATAGAGGAATTGCCCTCGGTGGAACAA
GAGTTTTGATGGTTTATCATGTTTGGTTGGAATTACAAAGTGGCTGTTATAAGGTTGGTTCTCCAACAAAAACTTTTCCT
TTCACTGTGAATAGGAAAAATTGTAAGTTCCAGCGATTAGGTCTGAAGGATCTTGTACTGATTGACTTTCCCCCAACTTT
TGTTTCTTTTCCTGTTTTAAAAATAGAGAAGTGGTTGCTATCATCCCATGATCCCTTTATGGCTGGCTCTGGTTGGTTTA
TGGAGATGCTCTTTCGGGAAAACGGGGTCGTTGAAGTAGCAAGAGAAGAAGCTGATTACACACTCTTGGATACTAACGAT
GTTTATGATGCAGCTTTTCTGAAAGGAGTTGGGTTGAACAAATGTGTGCGTTATACCATTTGTGATGATACTGGCACTGG
CTACCGGAATGATTTCTTCTATGTTTCCCAGTGTGGAACCCCTCTGGTCGCTAACTATGGGAGAGGGCGAGGGTTAAAAA
TTGCTTCAATTCATGTGGTTCACCATTTCTCTGCAACTGCTCGTGATACCATCATAGCAGGATCTGGTAGTCTTATCACG
AAGGAGGAATATCTTGAGGCAAGTTTGCTCCTAGGAGATGTCAAGCATCCTTTGGAAACAGACCGCATCCAGTCTTCTGG
TTGTTTGAGTGGTGAGGAATTTTTTGATGCTGAAACTGTCTTTCCAGAGGGTTTACTCACACCTGCTGAAGCTCCCAGAC
AAGCAACCTCTAGTGAGATTAGAAAAAGTTCAATATCTGCTGACTTGGAGTTGTTAACTGGAGAGAAGAGGAAAACAGAA
CCAGCTATTATAAGTAATAGGGATACTCGCCTTCATGATAAGAATTTGGACATTTTTAAAAAAGGCATGATGAAGTATAA
AGCAGTGGCTGCTGATATGAGTCCTGTTAACGATGAGGAGGAAAAGATCTGGAATCTCACTTGGGATAGTGTTTTTGATC
TGCCCGGGGGCATTGCTGGAAAGTGCCACCTTCTTAGTGAGGACGAGAATTTGAATGGTAGAGCTGGAGATAATGAGTAT
CGTGGCATGGTTGTCTCCACCAGTGAAGGTTGGCCTGAAGTCCTGAACAGAACAAATGGTGAAGCTGGCAAGGAGAGATT
TTTGCTTGGTTTGCCGGGTTGTTATACTTTGAATCGGGCCTTACCAATGTACCAAAGAATCTTAGATATGGACGCGCTTT
CTGAGACTACAATACCTTGCATTGTTGGACTTGATACAGCCAAAGATGAGCGGCTACCATTATCTAAAATATACCAGGAT
GTGAAAACAAGACTGTTCACAATATTACCCATGGAATATAACTACCTGGTGCGGAAATACTTTGGCTCTTTTGTTGCAGA
GCTAATGAAGCTACACAACTGTGTTCCAACAAAGGTGGGTATCAATCCATTGGGATATGATTGGACAATTCTTGGAAAGC
GGATGCATTCAAAAGGAACCAATTGGTTCAATGGGGATTATTCCCGCTTTGATGGAGTCACGCCACGCTGTTTGTTGATT
GAGATAGCTCATCGTATCACAGCTCTTTATGGAGATCAGCATGGAACTAGGCGTCTGCATTTAATGTTAGCTGCCACAAC
ACGATTGGGTGTTGCAGGAATAGGCCTATATCGTGTTTCTGGTGGTATCCCTTCTGGCTTTGCTTTGACAGTTATTGTCA
ACTCCCTTGTAAACCACTTCCTTGTCCGTTGGAGCTGGGAGAATATGATGGCAAGCTCTTCCCTTTTCTTTTCTGATTGT
GTAGAGTTGGCAGTTGTGGGTGATGATAATCTTGTCAGTGTGAAGCAGGTGGCTGCAGCAGACTTTAATTTAAGGAAGCT
TTCTGCTTTCTTGAAGAATTATGGCTTCACTTTGAAAGATGGTTCTGATAAGAATAAGGAAGAGCTGCCGGACTTTAATC
CACCAGAAAAATGTGACTTTCTGAAGCGGTGTTTCAAAGCAAGGGGTGACCGTTATTTGGCACCTCTCTCTTGGCTCTCT
CTTTCTGAGTCATTACATTGGGTGCGGGAGACCAACATGAGTAATGCCGCTGCTACGCAAAACAATGTTGAGGGCTTTTT
GCGTGAATTGTTTCATTACGGAGATAAAGAGTTGTATTGCAAGTGGAGGAGGGATCTTATAGAGTTGTGTGCTAGAAATA
GAGTTCCTTCTCCTGCTAGTTACACTTTTGAGGAGTTAGAGCGTGCTTGGCTTTCAGGGCGGACAGTAGCTTCGATTTTT
GAAAAAGAAGAGCCTGAGCTCATTGTTATCAGAGATGCAGCCTCTGATATCGCTCCTGATGTTCACATTGTACCTGTGCA
GCAATGTTTAAAGTGGAACTCAAGTGAAATGCCCTTAGTGGTGTGGTGTGGTCCCAATTGTCCAAATCAGCTTAAGAACT
CGAATAGCTGTTTTGCCATTCAGGCACCTCAGGGTTCCAAGTATCCTCTGCGCAATACGGTGCGCAATTTGCTGCGCAAA
GTGCACCAACGGGGTGACAAAGTCTACTTCACAGGCGCTCTAGACCAATCTTTGGTTCATTGGGTGGCTGCTTTTTATGC
TTCCATGTATCGTGACTCTTTTCATCATTCTGCTTATATGAAAGCATACTTTGGAGATGATGATAGGGGTTTGCTCGCTG
CAGTTACAGCTGCAAAAGGATGGTAGTTTTCTCTTAGTTTTGTTTTCTCCGGGGGTCTTCCATGCTTTCCTTAATTGGTT
GAGTGTGTTTGTTACACCCCCTTTGCTTTCTTTTGTTTCCATAAAGGATTAACCAGGCATCCTCCTACTCTGGTAGGTTT
AGTTCTGGCTTTAGAAAAGCTTTAAAAAAAAAAAAAAAAAAAAAAAAAAAAAAAAAAAAAAAAAAAAAAAAAAAAAAAAA
AAAAAAAAAAAA

CnVYV-A1_RNA2

CTCTGGTTTGAAAAGCAATCTGCGAACTTTGTTACTAATTTGTCTTCTCAACTAGCTATTGGGCTTATCCAATCTTAACG
ATCTTTCGCTCTTCTTGTTCTCTTATTTCTTTTGGTTGTGCTGCGGTTATACAGCTTTCCTCGTTGGAATTCAGGTCTTA

Additional file 3

ACCTGAACAACTGTTCTTTGTTCTGTTTGTCTCTCCGCATTAATTTTCCTTTGGAGTTTCAAACTTTTCACAACGTTTAC
ATTAAGCAACTTGAGTACCCCGATTTGTCAAGTTAGGGGTTTTTGTTTTTCCTACTTTCTTTCTCAGAGCACTATTGTTG
GTTTGTTTTTTCTTCTTTTACTATTTTATATTTGTATTCCTCCTTTTGTTTTCTGGATGGCTTCCTTCTTTTCTCGCCGG
TCACCAAAGGAAATGCCTAATGTTCCCTCCTATACTCCTAAGGAGATGGAGGTACTGAAAGCTGCCATTACTGAATGTGG
CATGAATTACGCTGATGTAGTGAGAGCTGCACAAACTGAGAGTGGCAAGATGGCCCTTCTCACTGCTGCAAACACTAGTC
AGCTGAAGAGTCTTGTTGCAACAACAGCAACGAGCAATTCTCTTGCCCGTTTGCGGGAGAAGCCTAAAGCTGTTGTTCAT
GTTTCTGAAGGGGTGGGTAGGCAAGTCCTTTGTGATGATAAGCTAACTCACCAAACTACTGTTGTTCATACTCCTGAGCC
CCTCTTTAAGAAGATGAAGGAGAAAAAACTTAAGAATGGTAAAGATACAGGGGGGGAAGAACCAAAATACCGTTCAGCTG
ATATTTGTACAATACAGCTTGGAGTGACCTCCCATCTCTCGGGCCATTCTAACCAAATAGCTGGTGTAGTTCTTCTGGAC
GGGAATAGGTCAACACAAGAGCAAGCTGTTCTCGGAATAGGGGTCCTTCCTCTTTATGAGGCACACTCTCATGCTCTGTT
TGCACCAAGGCTCAACGTCCACTATGATGATCCAAATTTTGTGGACCGATTACAGTTGTTAACTACCTTCTCAGATGATG
TTTTAGGGGGTGGCTCACCTGCTATGTCCTATAGTACCATTTCAGTTGTTAGGCATAATATAGCTGATGCCCATTACTTG
CCAGATCCACTACCTTATTCTAAGCTTAAAGAAAAATACCCTTGTGGTGTTCGGGGTCTCTGTAATTTTCAGGCTGCTGA
GATAGCGCCTCTACAACCTGCTCGTCTGACGCGTAGTGCCTCCTACATTCCTATACAACGTGCAGGAACTAAGACGGTTC
TAACCTTTGAGGATACAGTTGCTCAGGTGAAGGAACAGCAACCCATTCAGGCACGAGCTTCCTTCTCCTTTGCAAATGTT
TCATATGGTCAGCAGGACAGGGTGGCATTGGCTAGTAATTTACCACAGAAGTGCCAATTTCATAACAAGATAGGTTGCCC
TTGTGCTCAACATACTGGTAGAACTGAAGCAAGTGGGGACCACCATGAGGAGTTGGTTCCAGCTTCAGATGGTGGTACAG
AGGGACAGTTCTTTTCCCCCCAGCCTGTGGTGCATCCACCAGACTCTAAGTTTGTTGGTTCTCATCCCTTTTCTTTTCCG
GTAAACTCTAATGTTGGAACAGTTGTTTATACTTTACCGGTTATTCGAACTTCTTTGAAGGATACTGAGTATGGTAGGTT
TTATAAGGGATATCGCTACCTTCGGTGCAAACCTACTGTTCGATTAATTGGTTCAGGTCCCATACAGGCTAAGGGTCTTT
TGTGGCTTTGTTATGACCCTTCTGAAACTCTGGCTAAATATCCAAGTAGAGAGAGGGCTTTGGCTCTACAAGGAACTTGG
TTTATGCCTGGACGGCACGATTCGGCGTCCTTGACTGTTATGGAATTAGCAACTCCAGCTGGTTTTTGTGACATGGACAC
TGATGTCAATGGGGCCTTTAAGGTGGTTATTATTAAAAATTTGGCTAACTTTGACGTCACTGATTATGGTATGGAACTTT
CTTTGTATTTGGAAGTGGAAAGAATAGGCTTGGGTGATATAACTGTTGGTGGGGAATTGACGAGTTTCTATCCCTTGCGA
CAGATAGTACTTGACTATGAGTTGTCCACTACAACTTCTAAAGGGAAGGCCTTAGTTCTACCCTTGAACCCCTTGCTGCC
AGCTTGCGATGATGCCCAGTTTTATCCTAGCTGCTCTTCTTCCATTTTGGAGAATCATAGATATTGGAAAGGTACTCTTT
CGTTAGAAGTGATTTTCAATATGCCTGCTATGGCTGGTGGTATTGCTGAACTGGCCTTCGCTTATGATACTTATGACCAG
GCCGATGGTGACACCTACCGCCGTTTTGGTTCCTCTGTGGTTGACCTAAGGGCTCATCGTATATTAAGAGCTAGAGTGCC
TCTTAGTGGATATGGGGGTTATCTTGCTGGTGGTTCAGGCTCTCTTTTTGAGGTTAAGCCACAGACTGGTTTTGGGGACG
TCCTAAAATTAGTTATTTTGTTCACAGCCCCTCTCCATATAAGTGATACTTCAAAGAGAGGGTCTGTCCTTGTTAGGTAT
CTTGGTTTGGAGGACCTGGATTATCTAGAGCCAGCCACATCAATTGGGAGGTTGAATCCTAAAACCACCTTGGTACCAAA
TACAGCTGCTTCTGGGGGGCCTATCATAAAAGTAGGTACTGCAGAATGGGAGGAACCTTTTATAGCTCGAGTTCCCTTGG
GCTTGCGCCAGAAGACTTTTCGTTTGATGACAATAAACAAATGGTCTCCTTCTGGCTTTCTCTATTTTCCTGTTTCTCCT
GCGGTGCATTTACCACGAACTGCTGGTGGTTTCGAGGCTTCATTGGAGCAGCAATGCCCTCTGATGCATCGGAGCCAGGA
GAATTGTCAATGGAGGGGTACTTTGAGGTACCACTTGACTGCCCGTTTAGAGGGAGCTACACCGCAGTCAGTACTCCCCC
ATCGTTCTCTTACCTTCTCTGCTGTTCTCTTGAGTAAGATCCTTCCAGCACCCTGTTTTGTGGATAATAGCACTTTTAAA
CCACTACTTCCTTTGCTGTCAGTGAGGGATACTTTCTCTTTGGAGCAGGATCATCCCTTTGTAGAGTTCTCCACTCCCCC
AGGAAGATGGATAAATACCCACTTTGGTGCAACTGAACAGTACACTTGGCGTACTTGTCCCGTGTGGGTTCTCCTCCAGT
TTCCACCTAAAACTATGGCGCAATTGCATGTAAGGGACGTGTCCTTGTGGGTTGAACCAACTATAGAGTATAGACATCCT
ATGGGCGGTTTCCCATTAACTATTCCTGAACCTTCACCAGCGCCAAAGTACTTTTTTGAGAACACTTTTAATGTAGGTTG
AGTTATTTCTTTTCCTTTTTCTATTATGGATCTTCCATACCTGCTCTTATTAGTTTTTGTGGTTGTTGTATCCTATAATG
TTTTGTTGTTTTCTCTTGGGGTCTCCCATGCTTTCCTTAATTGGTTAAGTGTGTTTGTTACACCCCTTTGCTTTCTTTTG
TTTCCATAAAGGATTAACCAGGCATCCTCCTACTTTGGTAGGTTTAGTTCTGGCTTTGGAAAAGCTTTAAAAAAAAAAAA
AAAAAAAAAAAAAAAAAAAAAAAAAAAAAAAAAAAAAA

CnVYV-A2_RNA1

CCTTGTCTTCGTGCTTTCTGTTGTGCTGCGGTAATACAGCTTTCCTTCTGGAATTCAAGTCTTAACTTGAACAACATTTT

CTTTCCTTTATTTCTTCTTTCTTATCTTTTAGTAACCGCTTTAATTTTCCATGGGTTACTCAAGAGGTGCGAGGGGGGGT
TCTGATAAGGCAAAGCTTTGTGGGGTGTGCTTTGCTGTCTTTCCTGATGTTCAGGAGAGAAAAGCACACTATAGGAAGTA
CAACTGCCGCGATGCATCTGGCATCCAGGGTGGTTTGGTAGTCACTGGGAAATGTGCTGCCCAGAAAGAGAGAAATCTGA
GCTCCTACTATGAAACTAAGGAGCTTCTTGCTAATGAAGATGGCTCAATTGAACAGATTGAGACCCAATTCATGTCTGAG
TTGCTGGAGTTTACCTCTGCCTACCCAGAAGCAGGCTTTAGCTTTGGCACAATTAATGAGAAAGTGCCTAAAATAGGTGT
CTCTTATCCAGAGGCAATTTATCGTTCCTCGTTCTTCCATGCTGAGGAATATGCTAAGTGGCAATCACATAAACCTGGAG
CAGCTGTGAATTTTCTTGCTCCTGATTATGTTCCACTTGCTGATGCCTGTATGAAGGGCGCTCGTCTCGCCATCCCTAAT
GCTGGCCTTTGGGAGAGTAAGACGGATCGTACTGGTCTTGGTGGGGACGATATTAAATACCGTTTTGACAATGATGATCT
TTTTGCCAAGACCGATCAGGGACTTAGCAGATTTGTCTTTTCAGGCACTGTTAGATCTGAGCTTCCAGCTTTACGGACGG
TTCCAGATCTGACGCAAGCTGACATAAAGACTATGGCACATGCTCTTTCTGATGAGTGGGTTGAATGGATTTACTTTGAG
GAGGATTACGAGGAGTTTCTCTCGGATTCTTTTGAAGAATTCTATCCGGATATCCCAGAGCCTCTATTGGAAGAAATTTC
TAGCCCAGTAAGTGTTTTGGATTCAGCTAGCTCGTATCTTCCTGGTACTAAGTGGTGTTATGATTCAGACACAGAAGATT
ACCATCCTGTTGCGCCTATAATGAAGTATAGCAACAAAAAGTTTGGTTTGGACTTTGTTGATTATACTGACACGGAGTAT
TTGGAGGTGCCTACTGGTAAGGTTTATACCGAGACCTGGTTTGTAGATGGTCTGTTACCAGGTTTCTGGTTTTTGCGTGG
AACTCCTATAGATGAAGCAGTGGTGTGTGAGAAACTTCCGCTGATAGGGAAAACTCCCTTAGAGCTCATGATAGAGTACC
AAGTGCAACCTACTGGTGATCTCCTATTGGACATTCAAAGAGCTCTTTATGCTTATTATGAGCGCGTGGTGAAGCCCAAT
AAAAAATATTTTGATTTGAATGGGGTTCCCACATACCTTATTTTTGAAGATTGGAGGGCAGATTTGCGCTTTCATTTGCT
AGCACCATTTGTTCAAGGTAACCAACTGAAGCATATGATGGTGATCATGAACAGTCCGCTCCCAGGATGGCTTCTTGATT
TTCCAAAAAGGCGTAAGGGAGGTGGAGTGCAAGCTAGTGGGTGGAATCCCTTCCGCTATTTTTCAAAACAGGTGACACTG
GGCTTCCTGGATGGGATTATGGCGAAACTCCAAGAGGCACTTGGCCCAGTTATTTCTGCTCTTGGTTTTGTATGGGATTT
GATTATCAAAGCCAAGGATTATGTTTTTTCTTTGTTGGAGGATATCATTGCTAGGAAAGCAGATATCTTGAAGGCATTGC
TCCAACCCGTTCTCTATGTTTGTGGTTTCCTAACTTTCTTAGGGGCTATAAGGAGTCTCAAGTCTATTTTGGAACACCAT
AAAATAGCACTTGACATTCTTACAACAGCGGCTGTGGGGCTGGGTGTCTATGTCATGGTGACATTCCTAAAACAAGCTAA
CCTTGGGGCTATGAAGAGAGCCAGCAGGATTTGGGAATTGGTTTGTGGCTGGGATAAGCCAACTGAGATGAAACAAGCTG
ATGAAAAGATCCTACAGACATTAGTGGAAGAGCATCCAGACCAAGAAGAGTGGTTAAAGAGCTTACTGGCCTCCCCACAT
AGTGTGCCAGAGTGTGTTATAGATATGACACACATGAAGGATCTTTTAAATCCCAAGTGTGCCACTTTCCAGGAGGCTTT
TGCTTTGGGTTCTACAGAGGCCTCTTCTGCTGGTATGCTATTTGGTTCGGGATTTATATGGAAAATACTTCTTCTCCTTT
GCCCTCTTTCTATGTTTGGAGTCTCTAAAACATTGTCTTGTGCTAAGGATTTGATTACAATCCAAGGTGGTCAAGATGCT
GCTGGTAGGTTCTTCCAGGACGTGATTGGTGGCACCCAGGAAGTTTTTTACACTTTAACAGGTAGCAAGAATGAATTTCT
TGATTATATATATGCTACTGTAGGTGTAGATTTTCAGGCCTGGAGGGGAGAGGTTTTGGAATTAACAACAGCAACCCCAA
CCTCAATCTTCTTGGGACCACAGGAACGTTTGAAGCGTTTGAGAGCCTGTAAGGATAAAGCAGATCGGTTGATACTTCAG
ATGGATTCCCGGAAGGTCCCCGGAGCTTATATCACACATTTTAACAATTTGCTTCAAAGTCTCGATCGTGCTCTAGTGGA
GTGTCAGCAAGCCCTTTCTGTGGGTAAATGGCGGAAAACGCCTGCATGCATTTGGCTCTATGGAGACTCTCATGTTGGGA
AGTCAGTTTGCACCCAATACTTGATTGATGATGTTCTAGACTCCCTTGATTACGCTCAAACTGGGAGAGTTTTCTCCAGG
AATGGTTCAGACTCTTTTTGGTCTTGCTACAAAAATCAAAGTGCTATTTTGTATGATGATTTTGGGGCTGTTTCTGAGGG
TGGACACTTCGATGAGGCGGAGATAATACGCCTAATAGCTCCAGCTCCTCTCCCCTTAAATATGCCAAATTTGGAAGCAA
AGGGGAACACTTGCTGTACTTCTGATTTTGTCTTCATAACAGCAAATCAGGCTGGCCTAACACCATCTGCTGTCGTCCAT
TGTAAGAGAGCCTTTGAAAACCGAAGACTCATTTTAGCTGAGGTCACTGCTGTTGAGGGTAGTCGATACCGTGACAGGTA
CCGTTTTACTATCCACCAAAAGAATGAGTCATATGGGCGAGATGAGCGTTTCCCAGTTATGAATTATGAGCAGTTCCTCC
AATTTACAGTCAACCAGTGTAAGCACCATTTTGAGGAACAAGTGGACTTGAGAGATGCCCCACATACCCAGATCTTTGAT
GCAGCTGATCAGATTGCAGCAGAGGCTGAGGAGGGGTTCCAAACAGCTGGCATAGATATTCCCTTTTGCATGCTCAAAAG
TCTAGATGGGAATAAGATTACCTACTACTCCCAGAAGTTATTCAAGGAGGTTACTGAGGACCTTTCTCCTGAGGATGCTG
GTATCATTCGTGCTCGTTTAGACCCAGGTGTTATAACACAGAGATTAGAGGCGTTGCTGCCGAAGGCTGCTTTTACTAAT
CTGAGAACACAGGTTGGTTTAGGTGTTGATCCTTTTGAGACACCTAATCCCTATTTTAATTGCACTCAAAGGGCTAAAGT
TGTGGGCCGTTTTATGAGACCAAAAGTGTCAGAAGCACAAATGCAGGAGAACAAGAGAAGTTTCGGAGTAGTGGTTAAAG
AGTTGGTTCAGGGTGCATGCAAATCGCTATCAGAAGCACCTTTTCTGGTAAAACTTATTCTTGGTTTTGGAGCACTTTAT
TATGTGGGCTTACCTATCCTTAGTTGGCTCAAGGACCTATATTCAGCCCCTTCACTTCTTACTTTATCTTCTCTCGGTGT
TATCAGGGCTTCAGGTTCTCTTTCAAGCTCTCAAGACCAAGAAACGAGACGCACTGCCTCTGGTAGAGAGCGGAGGCGCT

ATCTTTTGGAAGCATCGGGTCCTGGGGCACTTGCAGCAAAGCCACAGGATGTTGAGACGGATCTCCGTTCCCTTTCCAAG
CATTTGGTTGGTTTTACCAGTATAGACTATCCAGATCACCACTACAGGGGGATTGCCCTTGGTGGGACAAGAATTTTGAT
GGTTTATCATGTTTGGTTGGAATTGCAGAACGGTTGTTATAAGGTTGGCTCTCCAACAAAAACTTTTCCTTTCACTGTAA
ATAAGAAGAATTGTAAGTTCCAACGACTGGGTCTGAAAGACCTTGTGTTGATTGATTTCCCTCCAACTTTCGTTTCTTTT
CCTGTCTTAAAGATAGAGAAGTGGTTGTTATCATCCCATGATCCCTTCATGGCTGGCTCTGGTTGGTTTATGGAGATACT
CTTCCGGGAAAATGGGGTCGTTGAAGTAGCCCGAGAAGAAGCTGATTACACACTTTTGGATACCAATGATGTTTATGATG
CAGCTTTTCTGAAAGGAGTTGGGTTGAACAAATGTGTGCGTTATACCATTTGTGATGATGCTGGCACCGGCTACCGAAAT
GATTTCTTTTATGTTTCTCAGTGTGGAACTCCTCTGGTTGCCAACTACGGGAAAGGGCGAGGATTAAAGATAGCTTCAAT
CCATGTGGTCCATCATTTCTCCGCAACTGCCCGTGACACCATTATAGCAGGATCTGGTAGTCTCATTACGAAGGAGGAAT
ATCTTGAGGCAAGTTTGCTTTTAGGAGATATCAAGCATCCTTTGGAAACAGACCGTATCCAGTCTTCTGGCTGTTTGAGT
GGTGAGGAGTTTTTTGATGCTGAAACCGTCTTTCCGGAGGGTTTACTTACACCTGCTGAAGCTCCTAGACAAGCAACTTC
TAGTGAAATTAAAAAGAGTTCAATATCCGCTGACTTGGAGCTATTGACTGGAGAAAAGAGGAAAACAGAACCAGCTATCA
TAAGTAACAGGGATACTCGCCTGCATGATAAAAACTTGGACATCTTTAAGAAAGGCATGATGAAGTATAAAGCAGTGGCT
TCTGATATGAGTCCCGTTACTGATGAGGAGGAGAAGGTTTGGAACCTCACTTGGGATAGTGTCTTTGATTTGCCTGGGGG
TATTGCTGGGAAGTGTCATCTTCTTAGCGAAGATGAAAATCTGAATGGTAGGGCTGGAGACAATGAGTATCGTGGTATGG
TTGTGTCCACCAGTGAGGGCTGGCCTGAAGTTCTGAATAGAACAAATGGTGAAGCTGGCAAAGAAAGATTTCTGCTTGGT
TTGCCGGGTTGTTACACTCTGAATAGGGATTTGCCAATGTATCAAAGAATTTTAGATATGGATGCTCTTTCCGAGACTAC
AATACCTTGTATTGTTGGACTTGATACAGCCAAAGATGAGCGGCTGCCATTATCTAAAATATACCAGGATGTGAAAACAA
GATTGTTCACAATATTACCTATGGAGTACAACTACCTGGTGCGGAAATACTTTGGCTCTTTTGTTGCAGAGCTAATGAAA
TTACATAATTGTATCCCAACAAAAGTGGGCATCAATCCGTTGGGATACGATTGGACAATCCTTGGGAAGCGGATGCACTC
AAAAGGAACTAATTGGTTCAACGGGGACTATTCCCGCTTTGATGGAGTCACACCGCGCTGTTTGTTGATTGAGATAGCTC
GCCGTATTACAGCCCTTTATGGAGATCAGCATGGAACTAGGCGTCTGCACTTAATGTTAGCTGCCACAACACGATTGGGT
GTTGCAGGAATAGGCCTATACCGTGTTTCTGGTGGTATCCCTTCTGGTTTTGCCTTGACAGTTATTGTCAACTCCCTTGT
GAATCACTTTCTTGTCCGTTGGAGTTGGGAGAACATGATGGCAAGTTCCTCTCTCTTCTTTTCTGATTGCGTGGAATTGG
CAGTTGTGGGTGATGATAATCTTGTCAGTGTGAAACAGGTGGCTGCAGCAGATTTTAATTTAAGGAAGCTTTCTGCTTTT
TTAAAGAATTATGGTTTCACTCTCAAAGATGGTTCTGATAAGAACAAGGAAGAGCTGCCAGACTTTAATCCACCAGAAAA
GTGTGACTTTTTGAAGCGGTGTTTTAAGGCAAGGGGTGATCGTTATCTGGCACCTCTTTCTTGGCTCTCTCTTTCTGAGT
CGTTGCATTGGGTGCGGGAAACTAATATGAGTAATGCTGCTGCCACACAAAATAATGTTGAGGGTTTTTTGCGTGAGTTG
TTCCACTACGGTGACAAGGAGCTGTACTGTAAATGGAGGAGAGATCTTATAGAGCTGTGTGCTAGAAATAGGGTTCCTTC
TCCTGCTAGTTACACATTTGAGGAGCTAGAGCGCGCTTGGCTTTCAGGTCGGACTGTAGCTTCGATTTTTGAAAGAGAAG
AACCCGAGCTCATTGTTATCAGGGATGCAGCTTCTGATATAGCTCCTGATGTTCACATTATACCTGTGCAGCAATGCTTA
AAGTGGAGCTCAAGTGAAATGCCCTTAGTGGTGTGGTGTGGTCCCAATTGTCCAAACCAGCTCAAGAACTCAAATAGCTG
TTTTACCATTCAGGCACCTCAAGGTTCAAAGTACCCTTTGCGTAATACGGTACGCAATTTGCTACGTAAAGTGCACCAAC
GGGGCGATAAGGTTTACTTTACAGGCGCTCTGGATCAATCCTTGGTTCACTGGGTGGCTGCTTTTTATGCTTCCATGTAT
CGAGATTCTTTTCATCACTCTGCTTATATGAAAGCGTACTTTGGAGATGATGACAAGGGTTTGCTCTCTGCGGTTACAGC
CGCAAAAGGATGGTAGTTCCCTCTTAGTTTCGCTTTCTCTTGGGGTCCCTCATGCTTTCCTTGATTGGTTAAGTGTGTTT
GTTACACCCCTTTGCTTTCTTTTGTTCCCATAAAGGATTAACCAGGCATCCTCCTACTTTGGTAGGTTTAGTTCTGGCTT
TAGAAAAGCTTTAAAAAAAAAAAAAAAAA

CnVYV-A2_RNA2

CATGTAACAACTGAGCAACACGGTCACCGCAGTCATTGAAAAGCAATCTGCGAACTTTGTTACTAATTTATTTCTTCAAT
TAGCTATTGGGCTTATCCAATTTTAACGATCTTTCGCTTTCCTTTTTCTCTTATTTCTTGGTTGTGCTGCGGTTATACAG
CTTTCCTTATTGGAATTCAGGTCTTAACCTGAACAACTGTTCTTTGTTCTGCTTGTCTCTCCGTATTAATTTTCCTTTGG
AGTTTCAAACTTTTCACAACGTTCACTTTAAGCAATTTGGATACCCCGATTTGTCAAGTTAGGGGTTTTTGTTTTTCTTA
TTTTCTCTCTCAGACTACCATTGTTGGTTTGTTCTTCTTTCTTTTACTATTGTATATTTGCGTTCCTCCTTTTATCTTTT
GGATGGCTTCTTTCTTTTCCCGCCGTTCACCAAAGGAGATGCCTAATGTTCCCTCCTACACTCCTAAAGAAATGGAAGTG
CTTAAAGCTGCCATTACTGAATGTGGCATGAACTACGCTGATGTGGTGAGAGCTGCACAAACTGAGAGTGGCAAAATGGC

Additional file 3

CCTTCTTACTGCTGCAAATACTAGTCAGCTTAAGAGTCTAGTTGCAACAACAGCAACAAGCAACTCTCTTGCTCGTTTGC
GGGAGAAGCCCAAAGCTGTTGTCCATGTTTCTGAAGGGGTGGGTAGGCAAGTCCTTTGTGATGACAAGCTAACTCATCAA
ACCACTGTTGTTCATACTCCTGAGCCTCTCTTTAAGAAGATGAAGGAGAAAAAACTCAAGAATGGTAAAGATACAGGAGG
GGAAGAACCAAAATACCGTTCAGCTGATATTTGTACAATACAACTTGGAGTAACCTCCCATCTCTCGGCCATTCCAACC
AGATAGCTGGTGTGGTTCTTTTGGATGGGAATAGGTCAACACAGGAACAAGCTGTTCTTGGAATAGGGGTTCTTCCTCTT
TATGAGGCACATTCTCATGCCTTATTTGCACCAAGGCTTAATGTCCACTATGATGATCCAAATTTTGTGGACCGGCTGCA
ATTGTTGACCACCTTCTCGGATGATGTTCTTGGGGGTGGCTCACCTGCTATGTCCTATAGCACCATTTCAGTTGTTAGGC
ATAATATAGCTGATGCCCATTATTTGCCGGACCCACTGCCATACTCCAAACTAAAAGAGAAATACCCTTGTGGTGTTCGA
GGTCTCTGTAACTTTCAAGCTGCTGAAATAGCACCTCTTCAGCCTGCCCGCTTGACGCGTAGTGCTTCCTACATTCCTAT
ACAACGTGCAGGGACCAAAACGGTCTTAACTTTTGAGGATACAATTGCTCAGGTGAAGGAACAGCAACCCATTCAGGCAC
GAGCTTCTTTCTCCTTTGCGAATGTCTCCTATGGTCAGCAGGACAGGGTAGCATTGACCAGTAATCTACCACAAAAGTGC
CAATTTCATAACAAGATAAGCTGCCCCTGCGCCCAACACACTGGTAGAGCTGAAGCAAGTGGGGACCACCATGAGGAGTT
AGTTCCAGCTTCAGATGGTGGTACAGAGGGACAGTTTTTCTCCCCCCAGCCTGTGGTACATCCTTCAGACTCCAAGTTTG
TTGGTTCTCATCCCTTCTCCTTCCCGGTAAATTCTAATGTTGGTACAGTTGTTTACACTTTACCAATTATTCGTACTTCT
TTGAAGGATACTGAGTACGGTAGATTTTACAAGGGATACCGCTACTTGCGGTGTAGACCTACTGTTCGATTGATTGGCTC
AGGTCCTATACAAGCTAAGGGTCTTCTGTGGCTCTGCTATGACCCTTCCGAAACTCTGGCTAAATACCCGAGTAGAGAGC
GGGCCTTGGCTCTACAAGGGACTTGGTTTATGCCCGGACGGCATGATTCGGCAACTTTGACTGTTATGGAGCTAGCTACT
CCAGCTGGCTTTTGCGATATGGACACTGATGTCAATGGGGCCTTCAAAGTAGTCATTATTAAGGACTTGGTTAATTTTGA
TGTCACCGATTATGGTATGGAGCTTTCTCTGTATTTGGAAGTGGAGAGGATAGGTTTGGGTGATATAACTATTGGTGGGG
AGTTGACTAATTTCTATCCCTTGCGGCAGATAGTACTTGATTATGAGTTGTCTACAACAACTTCCAAAGGGAAGGCTTTA
GTTTTGCCCTTGAATCCCCTGTTGCCAGCCCATGATGATGCTCAGTTTTATCCTAGCTGCTCTTCTTCCATTTTGGAGAA
TCATAGGTATTGGAAAGGAACTCTTTCGTTAGAAGTGATTTTCAATATGCCTGCCATGGCTGGTGGTATTGCTGAACTGG
CCTTTGCTTATGATACTTATGACCAGGCTGATGGTGACACCTATCGCCGTTTTGGTTCTTCTGTGGTCGATTTAAGGGCT
CATCGTATACTAAGAGCAAGAGTGCCTCTTAGTGGATATGGGGGTTATCTTGCTGGTGGTTCGGGCTCCCTTTTTGAGGT
TAGGCCGCAGACTGGTTTTGGGGATGTTCTAAAATTAGTTATTTTGTTCACAGCCCCTCTTCATATAAGTGATACCTCAA
AGAAAGGGTCTGTTCTTGTTAGATATCTTGGTTTGGAGAACTTGGATTATCTAGAGCCAGCCACATCGATTGGGAGACTA
AACCCTAAAACCACCCTGGTGCCAAATACAGCTGCCTCTGGGGGGCCTATCATAAAAGTGGGTACCGCAGAGTGGGAGGA
ACCCTTCATAGCCCGAGTTCCTTTGGGTTTGCGCCAGAAGACTTTTCGTTTGATGACAATAAATAAATGGTCCCCTTCTG
GCTTTCTTTACTTTCCTGTTTCACCTGCAGTTCATCTACCACGAACCGCTGGTGGTTTTGAGGCTTCATTGGAGCAGCAA
TGCCCCTTGATGCATCGGAGCCAGGAGAATTGTCAATGGAGGGGCACTTTGAAGTACCATTTGACTGCCCGTTTGGAGGG
AGCTACACAGCAGTCAGTACTCCCCCATCGTTCTCTTACCTTCTCTGCTGTTCTCCTGAGCAAAATCCTTCCAGCACCTT
GCTTTGTGGATAATAGCACTTTTAAACCATTACTTCCTTTATTGTCAGTGAGGGACACTTTCTCTTTGGAGCAAGATCAT
CCCTTTGTGGAGTTCTCCACTCCTCCAGGAAGATGGATAAATACCCACTTTGGTGCAAACGAGCAGTATACCTGGCGCAC
TTGTCCTGTGTGGGTTCTTCTTCAGTTCCCACCTAAAACTATGGCGCAATTGCATGTAAGGGATGTGTCTTTGTGGGTCG
AACCAACTATAGAGTATAGACATCCTATGGGTGGTTTCCCACTGACTATTCCTGAACCTTCACCAGCGCCAAGGTACTTT
TTTGAGAATACTTTTAATGTAGGTTAAATTTCCTTTTCCTTCTTTTGCTATGGATCTTCCACATCTACTTTTATTAGTTG
TTTGTGGTTGTTATATCCTACTTTCTTATTGTTTTCTCTTGGGGTCTTTCATGCTTTCCTTAATTGGTTAAGTGTGTTTG
TTACACCCCTTTGCTTTCTTTTGTTTCCATAAAGGATTAACCAGGCATCCTCCTACTTTGGTAGGTTTAGTTCTGGCTTT
AGAAAAGCTTTAAAAAAAAAAAAAAAAAAAAAAAAAAAAAAAAAAAAAAAAAAAAAAAAAAAAAAAAAAAAAAAAAAAAA
A

LycMoV-A_RNA1

ACGCAATCTGCGAACTTTGTTACTAATTTGTTTCCTCAACTAGCTATTGGGCTTATCCAATTTTAACAATCTTTCACTCT
CTTCTTTCAAGTGTTTCCTGTTGTGCTGCGGTTATACAGCTTTCCTTTTGGAATTCAAGTCTTAACTTGAACAACATTTT
CTTTCCTTCATTTCTTCTTTCTTATCTTTTAGTAACCGCTTTAATTTTCCATGGGTTATTCAAGAGGTGCGAGGGGGGGT
TCTGACAAGGCAAAGCTTTGTGGAGTGTGCTTTGCTGTCTTTCCTGATGTTCAGGAGAGAAAAGCACACTACAGGAAGTA

Additional file 3

TAACTGCCGTGATGCATCTGGCATCCAGGGTGGTTTAGTGGTCACTGGGAAATGTGCTGCCCAGAAAGAGAGGAATCTGA
GCTCCTATTATGAAACTAAGGAGCTTCTGGCTGGTGAAGATGGTTCAATTGAACAAATTGAGACCCAATTCATGTCTGAG
TTGCTGGAGTTTACCTCTGCCTACCCAGACACGGGCTTTAACTTTGGCACAGTCAATGAAAATGTGCCTAAAATAGGTGC
CTCTTACCCGGAGGCAATTTTTCGTTCCCCGTTCTTCCATGCTGAGGAGTACACTAAGTGGCAATCACATAAACCTGGAG
CAGCTGTAAACTTTCTTGCTCCTGACTATATTCCACTTGCTGATGCTTGTATGAAGGGCGCTCGTTTCGAAAAGCCCCAT
TCTGGCCTTTGGGAGAGTAGAAGGGATCGTACTGGTCTTGGTGGAGACGATATTAAATATCGTTTTGATAATGATGATCT
CTTTGCCAAGACTGATCAGGGCCTTAGCAGATTTGTCTTTTCAGGCACTGTTAGGTCTGAGCTTCCAGCTTTGCGGACGA
TTCCAGATCTAACGCAAGCTGATATAAAAACTATGGCATGCACTCTTTCTGATGAGTGGGCTGAATGGATTTATTTTGAG
GAAGATTACGAGGAATTCCTCTCGGATTCTTTTGGAAAATTTTATCCAGACATCCCGGAGCCTCTTCTGGAAGAGATTTC
TAGTCCAGTAAGTGTTTTGGACTCAGCTAGCTCGTATCTTCCTGGTACTAAGTGGTGTTATGATTCAGACACAGAAGATT
ACCACCCTGTTGCACCCATAATGAAGTATAGCAACAAAAAGTTTGATTTGGACTTTGTTGACTATACTGATACAGAGTAT
TTGGAGGTGCCTGCTGGCAAGGTTTATACTGAGACCTGGTTTATAGATGGTCTGTTGCCAGGTTTCTGGTTTCTGCGTGG
AACTCCCATAGATGAAGCGGTAGTATGTGAGAAACTTCCGCTGATAGGGAAAACTCCTCTAGAGCTCATGATAGAATACC
AGGTGCAACCCACTGGTGATCTTCTATTAGATATCCAAAAAGCTCTTTATGCTTATTATGAGCGTGTGGTGAAACCCAAT
AAAAAATATTTTGATTTGAATGGGGTTCCCACATACCTTATTTTTGAAGATTGGAGGGCAGATTTGCGCTTCCATTTGCT
AGCACCGTTTGTTCAAGGCAACCAATTGAAGCATATGATGGCGATCATGAACAGTCCGCTTCCGGGCTGGCTCCTTGATT
TCCCAAAAAGGCGTAAGGGAGGTGGGGTGCAAGCTAGTGGGTGGAATCCCTTCCGCTATTTCTCAAAACAGGTAACACTG
GGCTTTCTGGATGGGATTATGACGAAACTCCAAGAAGCACTTGGCCCGGTTATTTCTGCTCTTGGTTTTGTATGGGATTT
GATCATCAAAGCCAAGGATTATGTTTTCTCTTTGCTGGAGGATATCATTGCTAGGAAAGCGGATATCTTGAAGGCATTGC
TCCAACCTGTCCTTTATGTTTGTGGTTTCCTAACCTTTTTAGGGGCCATAAAGAGTCTCAAGTCTATCCTGGAACATCAT
AAAATAGCACTTGACATTCTTACGACAGCGGCTGTGGGGCTGGGTGTTTACGTCATGGTAACATTCCTAAAACAAGCTAA
CCTTGGGGCTATGAAAAGAGCCTCTAGAATTTGGGAGTTAGTTTGTGGTTGGGATAAGCCAACCGAGATGAAACAAGCTG
ATGAGAAGATCCTACAGACATTAGTGGAGGAGCATCCAGACCAAGAAGAGTGGTTGAAGAGCTTGTTGGCTTCTCCACAT
AGTGTGCCAGAGTGTGTTATAGATATGACGCATATGAAGGATCTTCTAAATCCCAAGTGTGCCACTTTCCAAGAAGCTTT
CGCATTAGGTTCTACAGAAGCCTCCTCTGCTGGCATGCTATTTGGTTCAGGATTTATATGGAAGATACTTCTTCTCCTTT
GTCCTCTCTCCATGTTTGGAGTTTCAAAAACACTGTCTTGTGCTAAGGATCTGATTACAATTCAAGGTGGTCAAGATGCT
GCTGGTAGGTTCTTCCAGGACGTGATTGGTGGTACTCAGGAAGTTTTCTATACTTTAACAGGTAGCAAGAATGAATTTCT
TGATTATATATACGCTACTGTAGGTGTGGACTTCCAGGCTTGGAGGGGAGAGGTTTTGGAATTAACAACAGCAACCCCAA
CCTCAATCTTTTTGGGGCCACAGGAGCGTTTGAAACGTTTGAGGGCTTGTAAGGATAAAGCAGATCGGTTGATACTCCAG
ATGGACTCCCGGAAAGTCCCAGGGGCTTATATCACACATTTTAATAACTTACTTCAAAGTCTTGACCGTGCTTTAGTGGA
ATGTCAGCAAGCTCTTTCTGTGGGTAAATGGCGGAAGACGCCTGCATGCATCTGGCTTTATGGGGATTCTCATGTTGGGA
AGTCAGTTTGTACCCAATATTTGATTGATGATGTTCTGGACTCTCTTGATTATGCTCAAACTGGGAGAGTCTTTTCCAGG
AATGGTTCAGATTCTTTTTGGTCCTGTTATAAGAACCAAAGTGCAATCTTGTATGATGATTTTGGAGCTGTTTCTGAGGG
TGGACACTTCGATGAGGCGGAGATAATACGCCTAATAGCCCCAGCTCCCCTCCCCTTAAATATGCCAAATTTGGAAGCAA
AGGGGAACACTTGTTGTACCTCTGATTTTGTTTTTATAACAGCTAATCAGGCTGGCTTAACACCATCTGCTGTCGTCCAT
TGTAAGAGAGCTTTTGAAAACCGAAGACTCATTTTGGCTGAAGTTACTGCTGTTGAGGGTAGTCGTTACCGTGATAGGTA
TCGTTTTACTATCCACCAGAAAAATGAGTCATATGGGCGAGATGAGCGTTTCCCAGTTATGAATTATGAACAGTTTCTCC
AATTCACAGTCAACCAATGCAAGCATCATTTTGAGGAACAAGTGGATCTGAGAGATGCCCCACATACCCAGATCTTTGAT
GCAGCTGATCAGATTGCGGCAGAAGCTGAGGAGGGGTTCAAGACAGCTGGAATAGATATTCCCTTTTGTATGCTCAAAAG
CCTAGATGGGAATAAGGTTACCTACTACTCTCAGAAGTTGTTCAAGGAGGTTACTGAAGACCTTTCTTCTGAGGATGCTG
GTATCATCCGTGCTCGCTTAGATCCAGGTGTTATAACACAGAGACTGGAGGCGTTGTTGCCGAAGGCTGCTTTCACTAAT
CTGAGAACACAGGTTGGTTTAGGTGTTGATCCTTTTGAGACACCTAACCCTTATTTCAATTGCACCCAAAGGGCTAAAGT
TGTGGGTCGTTTTATGAAACCAAAAGTGTCAGAAGCACAAATGCAGGAGAATAAGAGAAGTTTTGGAATGGTGGTCAAGG
AGTTGGTCCAAGGTGCATGTAAATCATTAGCAGAAGCACCTTTCCTGGTGAAACTTATTCTTGGTTTTGGAGCACTTTAT
TATGTAGGTCTACCTATCCTTAGCTGGCTCAAGGACCTATACTCAGCCCCTTCACTTCTTACCTTATCCTCTCTTGGTGT
TATCAGGGCTTCAGGTTCTCTTTCAAGTTCTCAGGACCAGGAAACGAGACGTACTGCCTCTGGCAGAGAGCGGAGGCGCT
ATCTTTTAGAAGCATCGGGTCCTGGGGCACTTGCAGCAAAGCCACAGGATGTTGAGACGGATCTCCGTTCCCTTTCTAAG
CATTTGGTTGGTTTCACCAGTATAGACTATCCAGACCACCACTATAGAGGAATTGCCCTCGGCGGAACAAGAATCTTGAT

GGTTTATCATGTTTGGTTGGAACTACAGAATGGTTGTTACAAGGTTGGTTCCCCAACAAAAACTTTTCCTTTTACTGTGA
ATAAGAAGAATTGCAAGTTCCAACGATTGGGTCTGAAAGACCTTGTGTTGATTGATTTTCCTCCAACTTTCGTTTCTTTT
CCTGTTTTAAAAATAGAGAAGTGGTTGTTGTCATCCCATGATCCTTTTATGGCTGGCTCTGGCTGGTTCATGGAGATACT
TTTCCGGGAGAATGGGGTCGTTGAAGTAGCCAGAGAAGAAGCTGATTATACACTCCTGGATACTAATGATGTTTATGATG
CAGCTTTTCTGAAAGGAGTTGGGCTGAATAAATGTGTGCGTTACACCATTTGTGATGATGCTGGTACTGGCTACCGGAAT
GATTTCTTTTATGTTTCCCAGTGTGGAACTCCTCTGGTTGCTAACTACGGGAAAGGGCGAGGATTAAAAATTGCTTCAAT
CCATGTGGTTCATCATTTCTCCGCAACTGCCCGCGATACCATTATAGCAGGATCTGGTAGTCTTATTACGAAGGAGGAAT
ATCTTGAGGCAAGCTTGCTTCTAGGAGATATTAAGCATCCTTTGGAAACAGACCGTATTCAATCCTCTGGTTGTTTGAGT
GGTGAGGAATTTTTTGATGCTGAGACTGTTTTTCCAGAGGGTTTACTCACGCCTGCTGAAGCTCCTAGACAAGCAACTTC
TAGTGAAATTAGGAAGAGTTCAATATCTGCTGACTTGGAGTTGTTGACTGGAGAGAAGAGGAAAACAGAACCAGCTATCA
TAAGTAACAGGGACACTCGCCTTCATGATAAGAATTTGGACATCTTTAAGAAAGGCATGATGAAGTATAAAGCAGTGGCT
TCTGATATGAGTCCCATTAGTGATGAGGAGGAAAAAGTTTGGAATCTCACTTGGGATAGTGTCTTTGATTTGCCAGGGGG
TATTGCTGGGAAGTGTCACCTTCTTAGTGAAGATGAGAATCTGAATGGTAGAGCTGGGGATAATGAATACCGTGGTATGG
TTGTGTCCACCAGTGAAGGTTGGCCTGAGGTTCTGAATAGGACAAATGGTGAGGCTGGCAAGGAAAGATTTTTGCTTGGT
TTGCCAGGTTGTTACACTTTGAATAGAGCCTTGCCAATGTATCAAAGAATTTTAGATATGGACGCTCTTTCCGAGACTAC
AATACCTTGTATTGTTGGGCTTGACACAGCCAAAGACGAGCGGCTGCCGTTATCTAAAATATACCAGGATGTGAAGACAA
GATTGTTTACAATATTACCTATGGAATACAACTACCTGGTGCGAAAATACTTTGGCTCTTTTGTTGCAGAGCTAATGAAA
TTACATAATTGCATTCCAACAAAGGTAGGTATCAATCCGTTGGGATATGATTGGACAATCCTTGGGAAGCGGATGCACTC
AAAAGGAACCAATTGGTTCAATGGGGACTACTCCCGCTTTGATGGAGTCACACCGCGTTGTTTGCTGATTGAGATAGCTC
GTCGTATTACAGCCCTTTATGGGGATCAGCACGGAACTAGGCGTCTGCACTTAATGTTAGCTGCCACAACACGATTGGGT
GTTGCAGGAATAGGTCTATACCGTGTTTCTGGTGGTATTCCCTCTGGCTTTGCCTTAACAGTTATTGTCAATTCCCTTGT
AAACCACTTCCTTGTCCGTTGGAGTTGGGAGAATATGATGGCAAGCTCCTCTCTCTTCTTTTCTGATTGCGTAGAATTGG
CAGTCGTGGGTGATGATAATCTTGTCAGTGTGAAGCAGGTGGCTGCAGCAGATTTTAATTTAAGAAAGCTTTCTGCTTTC
TTAAAGAATTATGGTTTCACCCTCAAAGATGGTTCTGACAAGAATAAGGAAGAGCTGCCAGACTTTAACCCACCAGAAAA
ATGTGACTTCTTGAAGCGGTGTTTCAAGGCAAGGGGCGATCGTTACTTGGCACCTCTTTCTTGGCTCTCTCTTTCTGAGT
CATTACATTGGGTGCGGGAAACTAATATGAGTAATGCTGCTGCCACACAAAACAATGTTGAGGGCTTTTTGCGTGAGTTG
TTTCACTACGGTGATAAAGAACTGTACTGCAAATGGAGGAGAGATCTTATAGAGCTGTGCGCTAGAAACAGAGTTCCCTC
TCCTGCTAGTTATACTTTTGAGGAGCTAGAGCGTGCTTGGCTTTCGGGTCGGACAGTAGCTTCGATTTTTGAAAAAGAAG
AGCCTGAGCTCATTGTTATCAGGGATGCAGCCTCTGATATAGCTCCTGATGTCCACATTGTACCTGTGCAACAATGCTTG
AAGTGGAACTCAGGTGAAATGCCTTTAGTGGTGTGGTGTGGTCCCAATTGTCCAAATCAGCTCAAGAACTCAAATAGCTG
TTTTGCTATTCAGGCACCTCAGGGTTCTAAGTACCCTCTGCGCAATACGGTACGCAATTTGTTGCGTAAAGTGCACCAAC
GGGGTGATAAGGTCTACTTTACAGGCGCTCTGGATCAATCTTTGGTTCATTGGGTTGCTGCTTTTTATGCTTCTATGTAT
CGGGATTCTTTTCATCACTCCGATTATATGAAAGCATACTTTGGAGATGATGATAAGGGTTTGCTCTCTGCGGTCACAGC
CGCAAAGGGATGGTAGTTTCCTCTTAGTTTTGCTTTCTCTTGGGGTCTCTCATGCTTTTCTTAGTTGATTAAGCGTGTTT
GTTACACCCCTTTGCTTTCTTTTGTTTCCATAAAGGATTAACCAGGCATCCTCCTACCCTGGTAGGTTTAGTTCTGGCTT
TAGAAAAGCTTTAAAAAAAAAAAAAAA

LycMoV-A_RNA2

CGGGAAAAGCAATCTGCGAACTTTGTTACTAATTTGTTTTCTCAACTAGCTATTGGGCTTATCCAATTTTAACGATCTTT
TGCTCTTCTTGTTTTCTTATTTCCTCTTGGTTGTGCTGCGGTTATACAGCTTTCCTTATTGGAATTCAGGTCCTAACCTG
AACAACTGTTCTTTGTTCTGTTTGTCTCTCCGCATTAATTTTCCTTTGGAGTTTCAAACTCTTCACAACGTTTACATTAA
GTAACTTGAATACCCCGATTTGCCAAGTTAGGGGTTTTTGTTTTTCTTATTTTCTTTCCCAGACCACTATTGTTGGTTTA
TTTTTCTCTCTTTTACTATTGTATATTTGTGTTCCACCTTTTGTTTTCTGGATGGCCTCCTTCTTTTCTCGCCGATCACC
AAAGGAAATGCCTAATGTTCCCTCCTATACTCCTAAAGAGATGGAGGTGCTGAAAGCTGCCATTACTGAATGTGGCATGA
ATTATGCTGATGTGGTGAGAGCGGCACAAACTGAGAGTGGCAAGATGGCCCTTCTCACTGCTGCAAACACTAGTCAGCTG
AAGAGTCTTGTTGCAACAACAGCAACGAGCAACTCCCTTGCTCGTTTGCGGGAAAAGCCTAAAGCTGTTGTTCATGTTTC

TGAAGGGGTGGGTAGGCAAGTCCTTTGCGATGATAAGCTAACCCACCAAACCACTGTTGTCCACACTCCTGAGCCTCTCT
TTAAGAAGATGAAGGAGAAAAAACTTAAGAATGGTAAAGACACAGGAGGGGAAGAACCAAAATACCGCTCAGCTGATATT
TGTACAATACAGCTTGGGGTGACCTCCCATCTTTCGGGCCACTCTAACCAAATAGCTGGTGTAGTTCTTTTAGATGGGAA
TAGATCAACACAAGAGCAAGCTGTTCTTGGAATAGGAGTCCTTCCTCTTTATGAGGCGCACTCGCATGCTTTGTTTGCAC
CAAGGCTCAATGTCCACTATGATGATCCAAATTTTGTGGACCGGTTACAATTGTTGACTACCTTCTCAGATGATGTTTTA
GGGGGTGGCTCACCTGCTATGTCCTATAGCACCATTTCAGTTGTTAGGCATAATATAGCTGATGCCCATTACTTGCCAGA
TCCACTACCCTATTCCAAGCTTAAAGAGAAATACCCCTGTGGTGTTCGGGGTCTCTGTAATTTCCAAGCTGCTGAGATAG
CGCCTCTGCAACCTGCCCGTCTAACGCGTAGTGCCTCTTATATTCCTATACAACGTGCAGGAACTAAGACGGTTTTAACC
TTTGAGGATACAGTTGCTCAGGTAAAGGAACAACAACCCATTCAGGCACGAGCTTCCTTCTCCTTTGCAAATGTTTCCTA
TGGTCAGCAGGACAGGGTGGCATTGGCCAACAATTTACCACAAAAGTGCCAATTCCACAATAAGATAGGTTGCCCCTGTG
CCCAACATACTGGTAGAACTGAAGCAAGTGGGGATCATCATGAGGAGTTGGTTCCAGCTTCAGATGGTGGTACAGAGGGA
CAGTTCTTTTCCCCCCAGCCTGTGGTACATCCACCAGACTCTAAGTTTGTTGGCTCTCATCCCTTTTCTTTTCCGGTGAA
CTCTAATGTTGGGACAGTTGTTTACACTTTACCGATTATTCGTACTTCTTTGAAGGATACTGAGTACGGCAGGTTTTACA
AGGGATATCGCTACCTGCGGTGTAAACCTACTGTTCGATTAATTGGTTCAGGTCCCATACAAGCTAAGGGTCTTCTGTGG
CTTTGTTATGACCCTTCTGAAACTTTGGCTAAATATCCAAGTAGAGAGAGGGCTTTGGCTCTACAAGGAACTTGGTTTAT
GCCTGGGCGGCATGATTCGGCGTCCCTGACTGTTATGGAATTAGCAACTCCAGCTGGCTTTTGTGACATGGACACTGATG
TCAATGGAGCCTTTAAGGTGGTTATTATTAAGGATTTGGCTAATTTTGATGTCACCGATTATGGTATGGAGCTTTCTTTG
TACTTGGAAGTAGAAAGGATAGGCTTGGGTGATATAACTGTTGGTGGAGAACTAACAAATTTTTATCCCCTGCGGCAGAT
AGTACTTGACTATGAGTTGTCTACTACAACTTCTAAAGGGAAGGCTTTAGTGCTACCCTTGAACCCCTTGCTGCCAGCTC
ATGATGATGCTCAGTTTTATCCTAGCTGTTCTTCTTCCATTTTAGAGAATCATAGATATTGGAAAGGAACTCTTTCGTTA
GAAGTGATTTTCAATATGCCTGCCATGGCTGGTGGTATTGCTGAACTGGCTTTTGCTTATGACACCTATGACCAGGCTGA
TGGTGATACTTACCGCCGTTTTGGTTCTTCTGTAGTTGATCTAAGGGCTCACCGTATATTAAGAGCTAGAGTGCCTCTTA
GTGGATATGGGGGTTATCTTGCTGGTGGTTCGGGCTCTCTTTTTGAGGTTAGGCCACAGACTGGTTTTGGGGACGTCCTA
AAATTAGTTATTCTGTTCACAGCCCCTCTCCATATAAGTGACACTTCGAAGAAAGGGTCTGTCCTTGTTAGGTATCTTGG
TTTGGAGGATCTGGATTATTTAGAGCCAGCCACATCAATTGGGAGGCTGAATCCCAAAACCACCTTGGTGCCAAATACAG
CTGCTTCTGGGGGGCCTATCATAAAGGTAGGTACTGCAGAATGGGAGGAACCTTTTATAGCTCGAGTTCCCTTGGGCTTG
CGCCAGAAGACTTTTCGTCTGATGACAATAAATAAATGGTCTCCTTCTGGCTTTCTCTACTTTCCTGTTTCTCCTGCGGT
TCATTTACCGCGAACTGCTGGTGGTTTCGAGGCTTCACTGGAGCAGCAATGCCCCTTAATGCATCGGAGCCAGGAGAATT
GTCAATGGAGGGGCACTTTGAGGTACCATTTGACTGCCCGTCTAGAGGGAGCTACATTGCAGTCAGTACTCCCTCACCGT
TCTCTCACCTTTTCTGCTGTTCTCTTGAGCAAAATCCTTCCAGCACCTTGTTTTGTGGATAATAGCACTTTTAAACCATT
ACTTCCTCTGCTGTCAGTGAGGGATACTTTCTCTTTGGAGCAGGATCATCCCTTTGTAGAGTTTTCTACTCCTCCAGGAA
GATGGATAAATACTCACTTTGGTGCAAGTGAACAGTATACCTGGCGTACTTGTCCTGTGTGGGTTCTCCTCCAGTTCCCA
CCTAAAACTATGGCGCAACTGCATGTAAGGGACGTGTCTTTGTGGGTTGAACCGACTATAGAGTACAGACATCCTATGGG
TGGTTTCCCATTAACTATTCCTGAACCCTCACCAGCGCCAAAGTACTTTTTTGAGAACACTTTTAATGTAGGTTGAGTTG
TCTCTTTTCCTTCTTCTATTATGGATCCTCCATACCTGCTTTTATTAGTTGTTTGTGGTTGTTGTATCCTACAATGTTTT
GTTGTTTTCTCCTGGGGTCTCCCATGCTTTCCTTCATTGGTTAAGTGTGTTTGTTACACCCCTTTGCTTTCTTTTGTTTC
CATAAAGGATTAACCAGGCATCCTCCTACTCTGGTAGGTTTAGTTCTGGCTTTAGAAAAGCTTTAAAAAAAAAAAAAAAA
AAAAAAAAAAAAAAAAAAAAAAAAAAA

CVX-A

CCAACACCAAACCAACTCACTCACACCTATCCAACTCAGAAGGGAGCTACCGTTACCGGTATATATTCTGTGGGATGGCT
CGTGTGCGTGAAGTTTTCTCATCGATCACCGACTCGTCGCTTAAAGCCGTAATTCAGGAGGAGGCCTATAAGACCATCCA
AACTCAATTACGACTCGCGGCCACAATCAACCCATACTCACAACCCACCGACGCTGCTGATGTTTTAGAGAACTTAGGAA
TTATCACTAACCCATTGGCCATTGAAGCCCATACCCATGGGGCGGCAAAGGCCATTGAAAATGACATGTATAACATAGTT
GCTAATTATTTGCCCAAGGAGAATCCAGTCACATTCTACTATATGAAGAAAGGCAAGCTCGGAAAAATTTAGGAGAGGCCC
ACACCAGAATGATCGTTTTGTCAACTCCCATTTTGAGCCCAAGGACATAGCCCGATACCCAGAGGAAACGGTTGTGGAAC

ACCTTGAGTCCACTCCCTGCACTACGAAGCTGGCTTTCATGGGCGACACTCTCCACTTCTGGACGCCAAAGCAACTACTA
ACTCTCTTTAACTTTAGTCCCAAACTAACCACTCTCTACGCTACTATAGTCTTACCTATAGAGGCTACACATAGACTACC
CTCTCTACACCCCGCCATATATACTCTGAAGTACTTCGGGGACTTCTTTATGTACATCCCTGGTGGACATGCAGGCGCCT
CCTACACTCATCACCAGAAACAACTAGCATGGCTTTTTGCCGGTCAAGTGTCTGGCCTAGGCATTAAGCTCACCATACAG
ATTCTGGAGAGCAAGGGTGCAAATCACCTGCTCATAATTCAAAGGGGTGATCTGCTCACTCCTCCCCTGAGGACTTTCGG
CTCTGACTCCCCCTATGTACAAATCCCACCCATCTTCCTCCCCAAAGACCATAATATGTCCCAGCCCATCCCCACGGTGT
TTGCAATGAAAATGTTCATGTACACCAAGTCCCTGAAAGAGGTCACTCCGCGTGATCTTTATGCGAAAATGAGACAGCTG
CTCCCGGACAAGGAACTGGCTAAATTCTCCCCAAGCCACATTGTCCACATGATGAACTATTTCTTCCTGCTCGGAAAATT
AGACTCTGTGAATCACTTTGAAAACTTGCTATCAGGGTCAATCGTCCGCAGGACATTTAAGCCACTCATCGTCTGGTGGC
AGCATTTTAAGGAAAAAATCAAGGGGCCCGCTGACTTTACAAAGATCTGCAAAGCGATTCAGTGGACCAATCTCGACCTC
ACATACAAAGTCCAATCCTTCACTCTCAGCACATGGGAGAATTTCTTCTCAAAGCAGATCACGCCTGAAGGGAAGAACGA
CGAGGACTTTTCGGACTTCTGGGATCCGAACTCCTACAACCCCTTAGACTCTGTGGATGATTTAAATGCTGAGCAGCTTA
AAGCCTGGGACTTCCTGCAGCAGCGCAACACCGGCGCCAACCCTGAGCCCAAATTCCATGACGATCCCCTTGAGAACAAG
TCTAACAAGGAAAAGTGCACTGCGGAACGAAGGGACTCCACTGAGGGCCCTCAGACCAATCAGGCCTCCTGCTCCACCCC
ACACAATGACTCGCAGGCCCCCAATGAAGAGAAACCAGAGGCGCCGCCCTACTTCACCTCACCCAAAGTTTTCACCACAC
CCCACGGGGTGGTGGTACGCTCTTTCCCCGCTGATCGCGACTGCGAGGAAACCTGGGAACATTTCATTATGGGCCATCTC
AGGCTGTGCGACAAAGTTGGTCAGAGGCAGGTCACTCTCTATGCCAGAAATGGAAAGATAAAGACCTACAGTTACGGAAA
TGTAACCCACAAGGCTCAACCCTGGCCCGCTGAGCTCACGGCCATCGCCAAAGCCCTTGATGTGCCTGAGGAGCACGACC
ACTGCCTCTTCCAGGTCTTCCAACAGGGAGCTGCAATCAACTTTCATTCTGACGACGAGCCACTGATTCAACCAGGCTCA
CCCATCACCACTCTTAGCATTGGGCATTGTGAACTGCTCACCAGGGATAAATCTACAAAAAAAATTCACAAGCAACTCCT
CTCCGGCCCCATCATCTACACCATGCCATCAGGCTTCCAGGAGACCCACCAACACTCAGTGCGCTCCCTACAAAAGAACC
GACTCTCCATCACTTTCCGCACCAGCGTGCACTGCAACCAAGAGGACACCCTCCCATGGCACAAGTGGCTGCCCATTCTG
AATGCAGCCGGGTTCAAAGGCAATCTGAGACAGGTCAATCCCAATGACGGGTCGCTCATTCTCCCAATCTCCGACATAAG
AAAACTGCCCACAATTGAAGCTGGTGACCCAGGCTTGCTAGAGGTACTCAAGAAAATCCATAGGAGTCCCACACCCTTCA
CATATGACCCACTCCGAGCCAAGGCTTTTGGTTCAGATGTCAAGAATCTCAGAGTAGGCGCCCTACTCCGACACCAGTCT
AAAGAATGGCTTGAGAGCTTTTCCAGAAGGACGGAGAAAGACAGCCGCTCTCTTGCTCTAACGGTCATTCATGGTGCTGG
GGGCAGCGGCAAGTCTCAGGCACTGCAGGACTTCCTCCGGGCCAACCCAGACAATCGCATAACGGTGGTCCTGCCCACCA
ATGAGCTGAGACTCGACTGGGTACGCAAACTGCCACAAGCCCATCCTTCCTCTCTCAAGACCTTTGAGAAGAGCCTGCTC
GCGCCAGCCAGTTCTACTACTATCATGGACGACTACTCCAAGCTTCCCGCGGGATTCATTGAGGCCTACATCGCAAACAA
CCCCGGACTGGAGTGTCTGATACTCACAGGGGATTCAAAGCAAAGCCACCACCATGAGCCTAACGACGGAGCCATGACCA
GCAAACTGTCGCCATTCACTGAGACCGCGTCACTCCACTGCAGATACTACCTCAATGCCACACACCGCAACAGGCAGGAC
CTGGCGAACATGCTCGGAGTCTACTCAGAGGTTGAGGGACGCACTAACATCACCATGGACACCACCATTCTTCCCGGCAG
GCATCTGCTCGTCCCATCCATGTTCAAGAAACAAGCATACGGGGAGTTAGGACACAAGGTCTCAACCTACGCCGGGTGTC
AAGGAATCACGGCGAATGAGATCCAAATACTGATTGACTCTGACACCCCAATGTGCTCCCAGCAGGTCATGTACACCGCC
CTCAGTAGGGCCGTGCATGCCATTCACTTCGTCAACACAGGGATAAACAATGATGCCTTCTGGAACAAGCTGGCAGCCAC
GCCCTATCTCGCGGCATTTCTGAGGTTGGTCCGGGAAGAGAAGACGAAAGAGCATCAACCTACAGAAGAGGGGCCCAGTG
CACCGCCCGCTCCCGAGACCCACTTCCCAGTGGAGAACGAGTCCACATTCTTTGACAAGGTCACAGACAACATGCCGGAG
AAGCATGAGAGGGAACTCTTTTCCACTCGCGATGGTTTCAGCAATTGCGTCCAGACTGAAGATCCCATCATCCAAATGTT
CCCCCACCAACAAGCAAAAGATGAGACTCTCTTCTGGGCAACAATTGAGGCTCGGCTGAAGATAACGAGCCCAGAGAAGA
ACTTCACTGAGTTCATCTCCAAGAAGCACATTGGGGACATTCTCTTCGAGAATTACAAGCTTGCCATGGGGCTCCCAAAA
GAACCAATCGCCTTCGACGAACGTCTTTGGAACTGTTGCGCGGACGAGGTGCAGAAGACCTACCTGTCTAAGCCACTGCA
CATGCTTCAAAACGGACAGGCTCGGCAGTCCCCTGACTTCGACCCCAAAATGATCAGCCTCTTCCTCAAGTCACAATGGG
TTAAGAAGATTGAGAAACTTGGCCAACCGCGCATTAAGGCTGGGCAGACCATCGCCTCTTTTCAACAAGAGGCAGTCATG
CTTTACGGAACAATGGCTCGATACATGAGGCGTGTCCGCGAGGTCTTTCAGCCTAAGAATATCATGATCAACTGCGAGAA
GACCCCTGAAGAGCTTACCAGCTGGGCAGCCGAGCACTGGAACTTCAATCGCAATTCATATGCCAACGACTACACGGCTT
TTGACCAGTCTCAGGACGGTGCGATGCTCCAATTTGAAATTCTCAAAGCTAGGCACCACTCTATCCCGGAAGTGTACATT
GAGGGCTACCTGGACTTGAAATGCTCATCCAAGACCTTCCTTGGCATACTGAAAATCATGCGACTCACTGGAGAGGGGCC
TACTTTCGACGCCAACACTGAATGCAACATCGCCTTCGCCCACACCAAGCTGAAAATACCAATCGGCACTGCTCAGCTCT

Additional file 3

ACGCGGGTGATGACTGCGCCTTTGACTACGCCCCAGAGGATAAGCCAAGCTTCAAAATGATCGAGACCGAGGTCTCCCTC
AAAGCCAAACCGGTCATCAAAAGGCAGGTCCAAGGGGAATGGGCAGAATTTTGTGGGATGTTAGTTACCCCCCTTGGCGT
CATCAAGGACCCCATCAAGACCTGGGCGGCACTGAAACTTGCCGAAAAGAGAGGCGACATGAAAGACATGAGGGATAGTT
ATGAAAGAGATGTCTGCCTTGCCTACCAGCACAAGGACGCTCTCCACTCAATCTTCTCAGAAGAGCAATCCATGGCCCAT
CAATTAACCGTTCGAAAGATCATCAAAGCAGGTGGAGGCCGTGTTTTCTCAACTTTTGACTGATGGAGACGCTAACTGCC
CTACTAATTGCTAACGGATACACTAGGACTGACTTGCCCATTTCAAAACCCTTAGTCATCCATGCTGTGGCCGGTGCAGG
AAAGACCACCCTGATTAGGCAATTTCTACACCAACACTCGGCCACGAATGCTCAGACGTTAGGCACGCCAGACAAGCCCA
ACCTCTCTAGGAAGATGATCCGACAGTTTTCAATGCCCAAGGCCAACCATTTCAACATCCTCGATGAGTACTGTGCACAG
CCTCTGAAAGGAAGTTGGGATGCGGTGTTCGCCGACCCTCTCCAGCATCCTGACTATGCCCTGGAACCCCATTTTATCAA
AGAGACCTCCCACCGACTTGGTCCAAGCACATGCGAGCTGATCTCCAGCCTTGGAATCCTCATTTATCCCGACTCAGAGG
ATCAAGTAGTCACAAGGAAAGGGGTTTTCGAGTCTGAACTCTTCGGTGTAGTCATAGCCCTTGACGAAGCCATTTTCCAA
CTCGCGAGCAGGCACGGACTCAAACCGCTCTGCCCCAAAGCAACCATTGGCCTCCAATTCCCTGTCGTGACAGTTCTATC
TTCTCTCGCCCTCGAGCAGGTTGAAGACAGTACAGCGCTCTACATTGCGCTTACGCGTCACACAAAAGAACTCCATGTCC
GGTGCCCCACTGAGGCTGACGCCACCACCTGACCACACTAGAATCCTCCTCCCACTAACCCTAGGCTTAGGACTATCGCT
AGTAGTATTCGCGCTGACTAGGTCCACATTACCCAGTGTCGGGGACTCTTCCCACAGTTTACCACACGGAGGCTGGTATC
GTGACGGCACGAAAACAGTCTTCTACTCAGGCCCTAAGAAAACGGCCATTAACTGGAGTCCACCCCTCTTTGTCTTTTTC
CTGACTTTAGCCATCTATGTTTCTTACCTATTTGAGTCTAGGAGTCGTGCTGGGCCTTGCAGTCATTGCGGGTCTAACCA
CACTTAACACCCCTAGTTGTCTGATCGTCTTGTCGGGTGACCAGACTGTTGTCAGGGGCTGTGAACACGTTGGCAATCTC
GCTGACGTCATCACGGCACTCAATAACCGCTTAAGTTTCAACAAATTGTGAAAATCAAAACTAATCATGTCTACTACTGG
AGTCCAGTCTTCTCAGTCCTCCGGACCCCGCTCCACTCCTCAGTCCGGACCTTTCCAAACCTTGTCCTCCTCCCAGCTTG
CGGCCCTCTCACTCGGGGTCACTAGCTCTCTTCTACCTTCCCCAGCTGAGCTAGTCAGCATCTCACAAGCCCTGACCACC
CTAGGCGCGTCTGCCACTAACCTCACTCCACTATCACTAGAAATAGTTAATTACTGCTTTGACAACGGGTCTTCACCAGA
AACAGTCTTCAAAGGTGACTCAACCGTGCTCCAGATGCCGCTCTCCAAAGTCGCCCATGCTATCACCCAATTCACCACTC
TGAGACAATTCTGCAGATACTTTGCGAAGATTATCTGGAATTATCGAGTTTCAAAGAATCTCCCTCCAGCGGCATGGGAA
GCCTGGGCCTACAAACCCGAGCAAAAGTTTGCTGCCTTCGACTTCTTCGACGGCGTTCTCAATGAGGCGGCCCTCAACCC
TACAGATGGACTTGTGCGTGTACCTAACGAGGCTGAACGACTCGCCAACCAAACTAATCGCAACGTCCATCTCTTTGAGA
GCAACGCACAAAAGAACAGAGCCCTTACCACTTCGGCTCTAGTCACCAAGGGGCTCCAAGGTTCAGAGTCTCCAAGGATT
CAATTTCTCCCAGGCCCTGAGTAGTTTAGAGATGAACAAGGCTCCCGCAGTAGCCTCCCACCGGGTTCCAACTGTGCCTA
TCCAGCTTAACTTAATAAGCCTAGCCTTCTGTTTTAATGCAAG
